# Efficiency enhancement of CZTSSe solar cells via screening the absorber layer by examining of different possible defects

**DOI:** 10.1038/s41598-020-75686-2

**Published:** 2020-12-11

**Authors:** Mehran Minbashi, Arash Ghobadi, Elnaz Yazdani, Amirhossein Ahmadkhan Kordbacheh, Ali Hajjiah

**Affiliations:** 1 Department of Physics, Tarbiat Modares University, P.O. Box 14115-175, Tehran, Iran; 2 Department of Physics, Politecnico Di Milano, Piazza Leonardo da Vinci 32, 20133 Milan, Italy; 3 Department of Physics, Iran University of Science and Technology (IUST), P.O. Box 16846-13114, Tehran, Iran; 4 Department of Electrical Engineering, College of Engineering and Petroleum, Kuwait University, Safat, 13133 Kuwait

**Keywords:** Electronics, photonics and device physics, Photonic devices, Electronic structure

## Abstract

This study represents the investigation of earth-abundant and non-toxic CZTSSe absorber materials in kesterite solar cell by using the Finite Element Method (FEM) with (1) electrical, and (2) optical approaches. The simulated results have been validated with the experimental results to define guidelines for boosting the cell performance. For improving the cell efficiency, potential barrier variations in the front contact, and the effect of different lattice defects in the CZTSSe absorber layer have been examined. Controlling the defects and the secondary phases of absorber layer have significant influence on the cell performance improvement. Previous studies have demonstrated that, synthesis of CZTSSe:Na nanocrystals and controlling the S/(S + Se), Cu/(Zn + Sn), and Zn/Sn ratios (stoichiometry) have significant effects on the reduction of trap-assisted recombination (Shockley–Read–Hall recombination model). In this work, a screening-based approach has been employed to study the cell efficiency over a wide range of defect densities. Two categorized defect types including benign defects (${N}_{t}<{10}^{16}$ cm^−3^ , N_t_ defines trap density) and harmful defects ${(N}_{t}>{10}^{16}$ cm^−3^) in the absorber bandgap in the CZTSSe solar cell, by analyzing their position changes with respect to the electron Fermi level (E_fn_) and the Valence Band Maximum positions have been identified. It is realized that, the harmful defects are the dominant reason for the low efficiency of the kesterite solar cells, therefore, reducing the number of harmful defects and also total defect densities lead to the power conversion efficiency record of 19.06%. This increment makes the CZTSSe solar cells as a promising candidate for industrial and commercial applications.

## Introduction

Thin-film photovoltaic (PV) solar cell, known as one of the great promises and flexible means for renewable energy science, has attracted tremendous research interests among scientists to develop it with high-efficiency absorbers to harness the solar energy^1–7^. Due to the mechanism of the solar cell, the two most essential reasons for choosing a material as an absorber layer are (1) the capability in absorbing light as much as possible to excite electrons to higher energy states and (2) the ability to move those excited electrons from the solar cell into an external circuit. Besides, as an essential point, choosing a non-toxic, environmentally friendly, and air-stable materials play a crucial role in manufacturing thin-film solar cells^6,8–10^.

In comparison to other photovoltaic solar cells, chalcopyrite Cu(In,Ga)(S,Se)_2_ (CIGS) and CdTe solar cells have attained great power conversion efficiencies (PCEs) of 22.6%^11^ and 22.1%^12,13^, respectively. Toxicity of Cadmium (Cd) and supply limitations for Indium (In) are barriers for the large-scale production of these solar cells. Therefore, the need for a non-toxic and earth-abundant material is the most critical standard to ensure an extensive low-cost PV solar cell development. Kesterite based materials including Cu_2_ZnSnS_4_ (CZTS), Cu_2_ZnSnSe_4_ (CZTSe), and Cu_2_ZnSn(S,Se)_4_ (CZTSSe), emerge as potential replacements for the chalcopyrite and CdTe absorbers^2,14–16^. Kesterite Cu_2_ZnSn(S,Se)_4_ (CZTSSe) solar cells have been seriously studied owing to their relatively high power conversion efficiency (PCE) with non-toxic and low-cost earth-abundant constituent elements. CZTSSe has been recognized as a prospective alternative absorber material due to its controllable bandgap (E_g_) (1.0 to 1.5 eV) and high absorption coefficient (more than 10^4^ cm^−1^). It should be noted that the bandgap (E_g_) of the CZTSSe absorber can be controlled by varying S/(S + Se) ratio from ~ 1.0 eV of Cu_2_ZnSnSe_4_ (CZTSe) to ~ 1.5 eV of Cu_2_ZnSnS_4_ (CZTS)^17–21^. Besides, variation of mixed powder of S/Se not only causes bandgap variation, but also changes the electron affinity (χ), absorption coefficient, and series resistance (the higher series resistance decreases the fill factor)^20,22–24^. The last confirmed PCE of 12.6% has been recorded for CZTSSe-based solar cell by using hydrazine based non-vacuum process^15^, and the PCE of 12.3% has been certified from vacuum-based process^20^. Recently, D.H. Son et al. recorded the PCE of 12.62% by studying the effect of solid-H_2_S gas reactions on the CZTSSe thin-film^25^. Cationic disorder and the presence of intrinsic defects are directly affecting the carrier concentration, electrical conductivity, elemental non-stoichiometry (which leads to the formation of unwanted defects and defect complexes) and other properties of CZTS, CZTSe, and CZTSSe films, as well as the performance of PV devices. Intrinsic point defects in kesterite structure may form during the CZTSSe thin-film/bulk crystal growth including vacancies (V_Cu_, V_Zn_, V_Sn_, and V_S(e)_), antisites (Cu_Zn_, Zn_Cu_, Cu_Sn_, Sn_Cu_, Zn_Sn_, and Sn_Zn_), and interstitials (Cu_i_, Zn_i_, Sn_i_, and S(e)_i_). Besides, the donor and acceptor defects may attract each other and form defect clusters or complexes (V_Cu_ + Zn_Cu_, 2Cu_Sn_ + Zn_Sn_, Zn_Sn_ + 2Zn_Cu_, 2Cu_Zn_ + Sn_Zn_…) because of their lower formation energy than antisite defects. To restrain high concentration of Cu_Zn_ antisite defects, which is a deep one, CZTSSe films have been developed with Cu-poor and Zn-rich (Cu/(Zn + Sn) ≈ 0.8 and Zn/Sn ≈ 1.2) phases. According to the similar ionic size (Cu, Zn ≈ 1.35 Å) and small chemical difference between Cu and Zn, the cation disorder is formed with the lowest formation energy. This cation disorder can induce electrostatic band fluctuation which leads a phenomenon called “V_OC_-deficit” which is the key factor that limits the cell efficiency^2,26–39^. For resolving this crucial problem, the crystallinity of the absorber layer, which depends on the annealing temperature, annealing time and sulfurization should be improved because impurity of crystallinity results shallow and deep defect levels within the bandgap, which is a detrimental effect on the cell efficiency^17,40–42^. Just like defects, the formation of complex secondary phases like Zn(S,Se) and Cu_2_Sn(S,Se)_3_ should be prevented because they all lead to structural inhomogeneity, local fluctuations of open-circuit voltage (V_OC_), and high recombination of photogenerated carriers in CZTSSe films, which negatively affect the efficiency of CZTSSe-based solar cells. All secondary phases have detrimental effects on the device performance because the lower optical bandgap of them reduces the open-circuit voltage (V_OC_) and fill factor (FF); while the higher bandgap may increase the series resistance and reduce the fill factor (FF) and short circuit current (J_SC_)^30, 43–47^. Also, a non-Ohmic back contact between the absorber layer and Mo causes high electron–hole recombination, which leads to notable open-circuit voltage (V_OC_) loss, and reduces the cell efficiency^2, 23, 48^. Therefore, (1) improving the crystallinity of the absorber layer, (2) preventing the formation of secondary phases, and (3) improving the quality of Mo/CZTSSe interface leads to impurity-free and phase-controlled CZTSSe thin films. These result an increment in the open-circuit voltage (V_OC_) and short-circuit current density (J_sc_), and with all these taken into account, it would be possible to record a better efficiency for the cell^30^.

A well-controlled heterojunction interface with fewer defects and secondary phases is an essential point to enhance the device performance, therefore, we try to take defects and unwanted phases under control by the simulation to improve the cell PCE. Characteristics of harmful defects can be altered by double cation substitution which is a feasible strategy to reduce the negative effects of the defects. The effects of double cation substitution by partially substituting of Cu with Ag and Zn with Cd have been explored on Cu_2_ZnSnS_4_ (CZTS) devices with 10.1% efficiency by Hadke^49^. It is shown that in substitution of Cd and Ag, Cd improves the cell performance by altering the defect characteristics of acceptor states close to the valence band, and Ag reduces non-radiative bulk recombination^49^. Recently, in order to reduce cation disorder in CZTSSe, isoelectronic cation substitution of Cd for Zn^37^ Ba for Zn^50,51^, Ag for Cu^52,53^, and Sn with Ge^54,55^ have been conducted. The isoelectronic cation substitution with larger ionic size, mismatching between cations, presents an approach, reducing cationic disorder with fewer defects leads improved solar cell performance^56^. To control the absorber defects, some approaches have been proposed, including: (1) synthesizing CZTSSe:Na nanocrystals^57^, (2) controlling the S/(S + Se), Cu/(Zn + Sn) and Zn/Sn ratios (Control Stoichiometry)^24,36,58^. Moreover, we propose adding Graphene^59^ and Graphyne to the absorber precursors in order to enhance the conductivity which improves the total mobility and reduces the defects.

In this study, the device simulations are examined using the Finite Element Method (FEM) in a 2D symmetric cylindrical coordinate. The simulated results have been validated with the experimental results to define guidelines for boosting the cell performance. Also, the cell efficiency has been investigated by studying the potential barrier effect in front contact and the connected semiconductor layer. In the next step, by identifying harmful defects in bandgap, we suggest a profitable way for improving the cell performance by analyzing defect position changes, monitoring the electron Fermi level (E_fn_) and the Valence Band Maximum (VBM) positions.

According to the results, the farther position of the defect from the electron Fermi level (E_fn_) records a better cell efficiency (19.06%, which is remarkable efficiency in comparison with other calculations). Besides, minimizing defects density causes an improvement in cell performance. Identifying the harmful and benign defects, and reduction in defect density helps us to record the PCE of 19.06%.

## Results and discussion

### Cell structure

**Figure 1:**
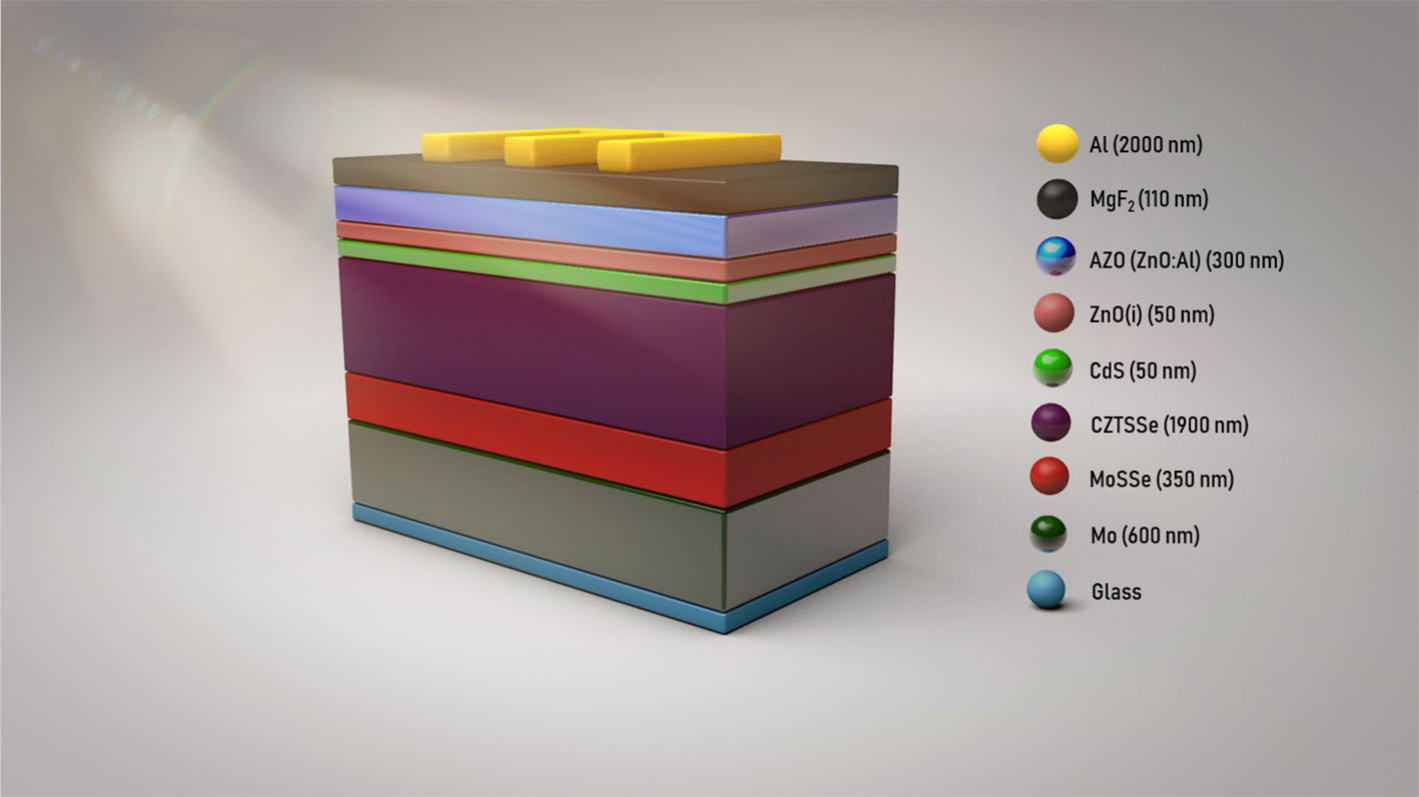
Schematic image of CZTSSe solar cell layers.

Based on the experimental model, the solar cell structure has been demonstrated in Fig. 1.

Table S1 (supporting information) represents electrical and optical properties of simulated layers in grading mode using for the simulation of the CZTSSe-based solar cell. Likewise, the basic data for defect concentration, the defect properties at the interface of graded CZTSSe/CdS, and for graded absorber layer are available in Tables S2, S3, and S4 in the supporting document, respectively.

### Defects and secondary phases control

1. Synthesizing CZTSSe:Na Nanocrystals

The presence of undesirable defects in the absorber layer during the deposition process, and formation of secondary phases, limit the cell performance by reducing open-circuit voltage (V_OC_). Synthesizing CZTSSe:Na nanocrystals is a method to overcome this problem. In fact, Na ions reduce concentration of deep level recombination, which results enhancement in the carrier concentration and open-circuit voltage (V_OC_), as well as the device performance^30,57^.

(2) Controlling the S/(S + Se), Cu/(Zn + Sn) and Zn/Sn ratios (Control Stoichiometry)

As we mentioned above, the CZTSSe bandgap is controllable by varying S/(S + Se) ratio from ~ 1.0 eV of CZTSe to ~ 1.5 eV of CZTS. The greater bandgap demonstrates more S/(S + Se) ratio in the film. The graded bandgap implies that different S/(S + Se) ratios of the absorber are formed by different sulfo-selenization temperatures. Higher sulfo-selenization temperature leads to more S^17^. Moreover, the Cu-poor, Zn-rich, Cu/(Zn + Sn) ≈ 0.80, and Zn/Sn ≈ 1.20, conditions are desirable for higher device performance. The high concentration of defect clusters or complexes results a notable non stoichiometry, which leads to lower device performance. Sulfurization at high temperature during the post-deposition helps to control the stoichiometry^24,36,58^.

### Numerical modeling

In this work, the CZTSSe solar cell has been investigated using the Finite Element Method (FEM), and validated by the experimental results. The simulation contains (1) electrical, and (2) optical sections, in a two-dimensional cylindrical coordinate based on charge carrier transport equations. In the provided structure, there is no nanoparticle, and due to the morphological properties of the absorber, the Beer–Lambert law has been employed to study the optical properties of the cell. The study of the electrical properties is divided into three parts, in which two parts are based on the Drift–Diffusion equations describing charge carriers with respect to the effect of diffusion and drift carriers, and also recombination rate. Furthermore, the third part is the electrostatic potential based on the Poisson equation^60–63^. The generation rate is calculated in optical part, which is available in the supporting.

### Charge carrier transport equations in two-dimensional symmetrical cylindrical coordinate (3D form with symmetry in the angular direction)

The Poisson and charge carrier transport equations were used for validation of the experimental kesterite solar cell case. Three-dimensional form of these equations are represented below:

1 $$\nabla \left[-{D}_{n}\nabla n+n{\mu }_{n}\left(\nabla\Phi +\frac{\nabla \chi }{q}+\frac{{K}_{B}T}{q}\nabla \mathrm{ln}{N}_{c}\right)\right]=g\left(\lambda ,r,z,\varphi \right)-U$$

2 $$\nabla \left[-{D}_{P}\nabla P-p{\mu }_{P}\left(\nabla\Phi +\frac{\nabla \chi }{q}+\frac{\nabla {E}_{g}}{q}-\frac{{K}_{B}T}{q}\nabla \mathrm{ln}{N}_{v}\right)\right]=g\left(\lambda ,r,z,\varphi \right)-U$$

3 $${\nabla }^{2}\Phi =\frac{q}{{\varepsilon }_{0}{\varepsilon }_{r}}\left(n-p-C\right)$$

where $n$ and $p$ are concentration of electrons and holes, ${D}_{n}=\frac{{{\mu }_{n}K}_{B}T}{q}$ and ${D}_{p}=\frac{{{\mu }_{p}K}_{B}T}{q}$ are electron and hole diffusion coefficients. ${\mu }_{n}$ and ${\mu }_{P}$ are electron and hole mobilities, ${K}_{B}$ is the Boltzmann constant, $T$ is temperature $q$ is electronic charge. $\Phi (F=-\nabla\Phi )$ is the electrostatic potential, χ is the electron affinity, E_g_ is the bandgap of the semiconductor, ${N}_{c}={\left[\frac{{m}_{c}{K}_{B}T}{2\pi }{h}^{2}\right]}^{1.5}$ and ${N}_{v}={\left[\frac{{m}_{v}{K}_{B}T}{2\pi }{h}^{2}\right]}^{1.5}$ are effective density of states of the conduction and the valence band, $h$ is the Planck constant,${m}_{c}$ and ${m}_{v}$ are effective mass of states of the conduction and the valence band. $g$ is the generation rate of charge carriers, λ is wavelength, and $U$ is the recombination rate of charge carriers. Moreover, ${\varepsilon }_{0}$ and ${\varepsilon }_{r}$ are vacuum and relative permittivity, $C={N}_{D}(\mathrm{donor density})-{N}_{A}(\mathrm{acceptor density})$ is the impurity density.

The $U$ equation depicts the sum of Shockley–Read–Hall, radiative, and the Auger recombination terms which described as:

4 $$U={U}_{SRH}+{U}_{rad}+{U}_{aug}$$

${U}_{SRH}$, ${U}_{rad}$, and ${U}_{aug}$ define as:

5 $${U}_{SRH}=\frac{np-{n}_{i}^{2}}{{\tau }_{n}\left(p+{p}_{t}\right)+{\tau }_{p}(n+{n}_{t})}$$

6 $${U}_{rad}={B}_{rad}\left(np-{n}_{i}^{2}\right)$$

7 $${U}_{aug}=\left({C}_{n}^{^{\prime}}n+{C}_{p}^{^{\prime}}p\right)\left(np-{n}_{i}^{2}\right)$$

where ${\tau }_{n}$ and ${\tau }_{p}$ are electron and hole lifetimes, ${n}_{t}$ and ${p}_{t}$ are electron and hole concentrations of the trap state. It should be noted that the strongest ${U}_{SRH}$ occurs when ${n}_{t}={p}_{t}={n}_{i}$. The ${n}_{i}{=[{N}_{c}{N}_{V} exp(-q{E}_{g}/{K}_{B}T)]}^{1/2}$ is the intrinsic carrier concentration, ${B}_{rad}$ is the coefficient of bimolecular radiative recombination , and ${C}_{n}^{^{\prime}}$ and ${C}_{p}^{^{\prime}}$ are electron and hole Auger coefficients. The Auger and radiative recombination are neglected due to their low effect on the kesterite cell performance^32,64^. In this study, defect distribution is investigated based on Explicit Trap Distribution (ETD), which is close to Shockley–Read–Hall (SRH) recombination statistics. The equations are written as:

8 $${U}_{ETD}=q{(R}_{n}-{R}_{p})$$

9 $${R}_{n}=\sum_{i}{{C}_{n}}^{i}{{N}_{t}}^{i}\left[n-n{{f}_{t}}^{i}-\frac{{{n}_{1}}^{i}}{{{g}_{D}}^{i}}{{f}_{t}}^{i}\right]$$

10 $${R}_{p}=\sum_{i}{{C}_{p}}^{i}{{N}_{t}}^{i}\left[p{{f}_{t}}^{i}+{{g}_{D}}^{i}\left({{p}_{1}}^{i}{{f}_{t}}^{i}-{{p}_{1}}^{i}\right)\right]$$

where ${U}_{ETD}$ is the ETD recombination rate, ${R}_{n}$ and ${R}_{p}$ are recombination rate of electrons and holes, ${N}_{t}$ is defect carrier density, ${g}_{D}$ is degeneracy factor, and ${C}_{n}$ and ${C}_{p}$ are electron and hole trap probabilities, which can be derived from following equations:

11 $${C}_{n}=\langle {\sigma }_{n }\rangle {v}_{n}^{th}$$

12 $${C}_{p}=\langle {\sigma }_{p }\rangle {v}_{p}^{th}$$

where $\langle {\sigma }_{n}\rangle$ and $\langle {\sigma }_{p}\rangle$ are electron and hole trap cross sections, ${v}_{n}^{th}$ and ${v}_{p}^{th}$ are thermal velocity of electrons and holes, and as we mentioned above, $n$ and $p$ are concentration of electrons and holes, which can be calculated with the following equations:

13 $$n={\gamma }_{n}{n}_{i,eff}{e}^{\left(\frac{{E}_{fn}-{E}_{i}}{{K}_{B}T}\right)}$$

14 $$p={\gamma }_{p}{n}_{i,eff}{e}^{\left(\frac{{{E}_{i}-E}_{fp}}{{K}_{B}T}\right)}$$

where ${\gamma }_{n}$ and ${\gamma }_{p}$ constants are related to the Maxwell–Boltzmann distribution function, and we assume they are equal to one (${\gamma }_{n}$ = ${\gamma }_{p}$ = 1). Also, ${E}_{fn}$ and ${E}_{fp}$ are electron and hole Fermi level energy respectively, and ${n}_{i, eff}$ is the inherent density, which is calculated by:

15 $${n}_{i, eff}={({N}_{c}{N}_{v})}^{1/2}exp\left(-\frac{{E}_{g}}{2{K}_{B}T}\right)$$

also, subscript “i” in Eq. 5 and Eq. 6, is related to the defect’s charge, which is categorized in the Table 1 below:

**Table 1:** Defect types with respect to i subscript in Eqs. (5) and (6)^61,65^.

Trap type	Trapped species	Occupied charge	Unoccupied charge	${N}_{t}^{i}$
Donor trap	Electron	0	+	${N}_{t}^{D}$
Acceptor trap	Hole	0	−	${N}_{t}^{A}$
Neutral electron trap	Electron	−	0	${N}_{t}^{n}$
Neutral hole trap	Hole	+	0	${N}_{t}^{p}$

It should be noted that, the defect energy difference is calculated by the following equation:

16 $${\Delta E}_{t}={E}_{t}-{E}_{i}$$

where ${E}_{i}$ is the reference energy, based on our assumption, it is ${E}_{i}= {E}_{v}$. (valence band position).

The final changing rate of trapped electrons derived by the following equation:

17 $${\mathrm{N}}_{t}\frac{\partial {f}_{t}}{\partial t}={C}_{n}{\mathrm{N}}_{t}\left[n-n{f}_{t}-\frac{{n}_{1}}{{g}_{D}}{f}_{t}\right]-{C}_{p}{\mathrm{N}}_{t}\left[p{f}_{t}+{g}_{D}\left({p}_{1}{f}_{t}-{p}_{1}\right)\right]$$

where ${n}_{1}$ and ${p}_{1}$ are calculated by:

18 $${n}_{1}={\gamma }_{n}{n}_{i,eff}{e}^{\frac{{\Delta E}_{t}}{{K}_{B}T}}$$

19 $${p}_{1}={\gamma }_{p}{n}_{i,eff}{e}^{-\frac{{\Delta E}_{t}}{{K}_{B}T}}$$

### Experimental validation

We studied the effect of optimization of various parameters for boosting the cell efficiency. Table 2 represents pervious reported performance parameters of Cu_2_ZnSn(S,Se)_4_ (CZTSSe) based solar cells with Cu/(Zn + Sn) and Zn/Sn ratios, which have been described as variable chemical components.

**Table 2:** reported performance parameters (V_OC_, J_SC_, FF, and η) of Cu_2_ZnSn(S,Se)_4_ (CZTSSe) based solar cells with Cu/(Zn + Sn) and Zn/Sn ratios, which described as variable chemical components.

Component	Cu/(Zn + Sn)	Zn/Sn	V_OC_ (V)	J_SC_ (mA/cm^2^)	FF (%)	η (%)
Cu_2_ZnSn(S,Se)_4_^66^	0.925	1.0	0.622	15.87	60.0	5.90
Cu_2_ZnSn(S,Se)_4_^67^	0.79	1.11	0.420	30.40	52.70	7.20
Cu_2_ZnSn(S,Se)_4_^68^	0.90	1.10	0.497	20.0	54.32	5.40
Cu_2_ZnSn(S,Se)_4_^69^	0.80	1.22	0.5627	24.07	60.0	8.13
Cu_2_ZnSn(S,Se)_4_^70,71^	0.80	1.20	0.516	28.60	65.0	9.66
Cu_2_ZnSn(S,Se)_4_^33^	0.80	1.20	0.517	30.80	63.70	10.10
Cu_2_ZnSn(S,Se)_4_^24^	–	–	0.4598	34.50	69.80	11.10
Cu_2_ZnSn(S,Se)_4_ (experimental case-this work)	0.80	1.20	0.501	35.34	65.62	11.62

Furthermore, optimized parameters have been investigated in the following sections.

**Figure 2:**
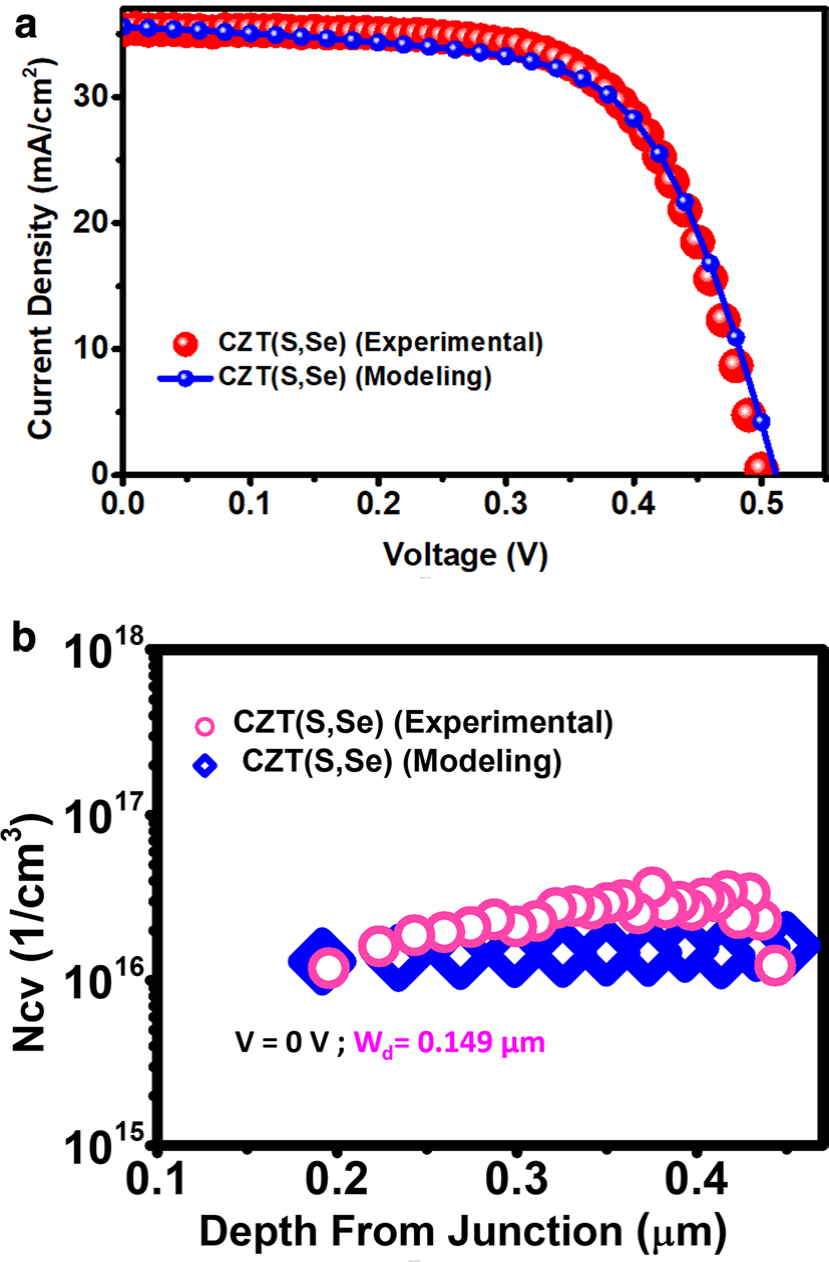
(**a**) Current density versus voltage curves for experimental and simulation results. (**b**) The C–V charge densities (N_CV_) in the CZTSSe absorber layer at temperature of 300 K and frequency of 100 kHz.

At first, for demonstrating the accuracy of simulated results, the comparison between the reported experimental data and the simulated one has been made. CZTSSe solar cell has been simulated using data listed in Tables S1–S4. The current–voltage characteristics (J–V) of the cell (Fig. 2a) indicates a good agreement between the experimental and simulation results.

The C–V charge densities (N_CV_) in the CZTSSe absorber layers at a temperature of 300 K and a frequency of 100 kHz has been measured (Fig. 2b). The depletion width (W_d_) at V_bias_ = 0 V was 0.149 µm for CZTSSe cell. By fitting the N_CV_, carrier density and epsilon of the CZTSSe layer have been extracted from experimental measurement.

### Optimization of the potential barrier effect in front contact

**Figure 3:**
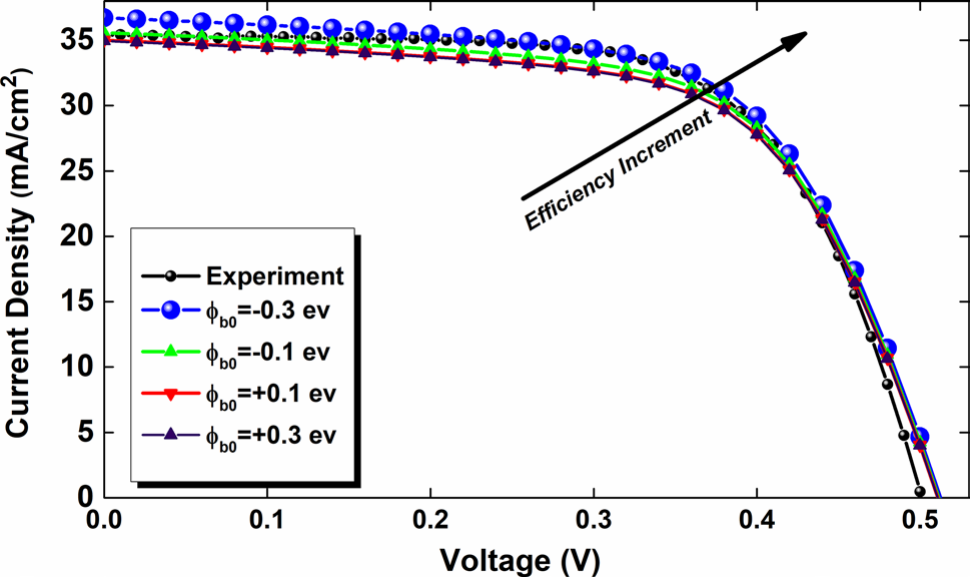
Current density versus voltage curves of the cell by optimization the potential barrier effect in front contact.

**Figure 4:**
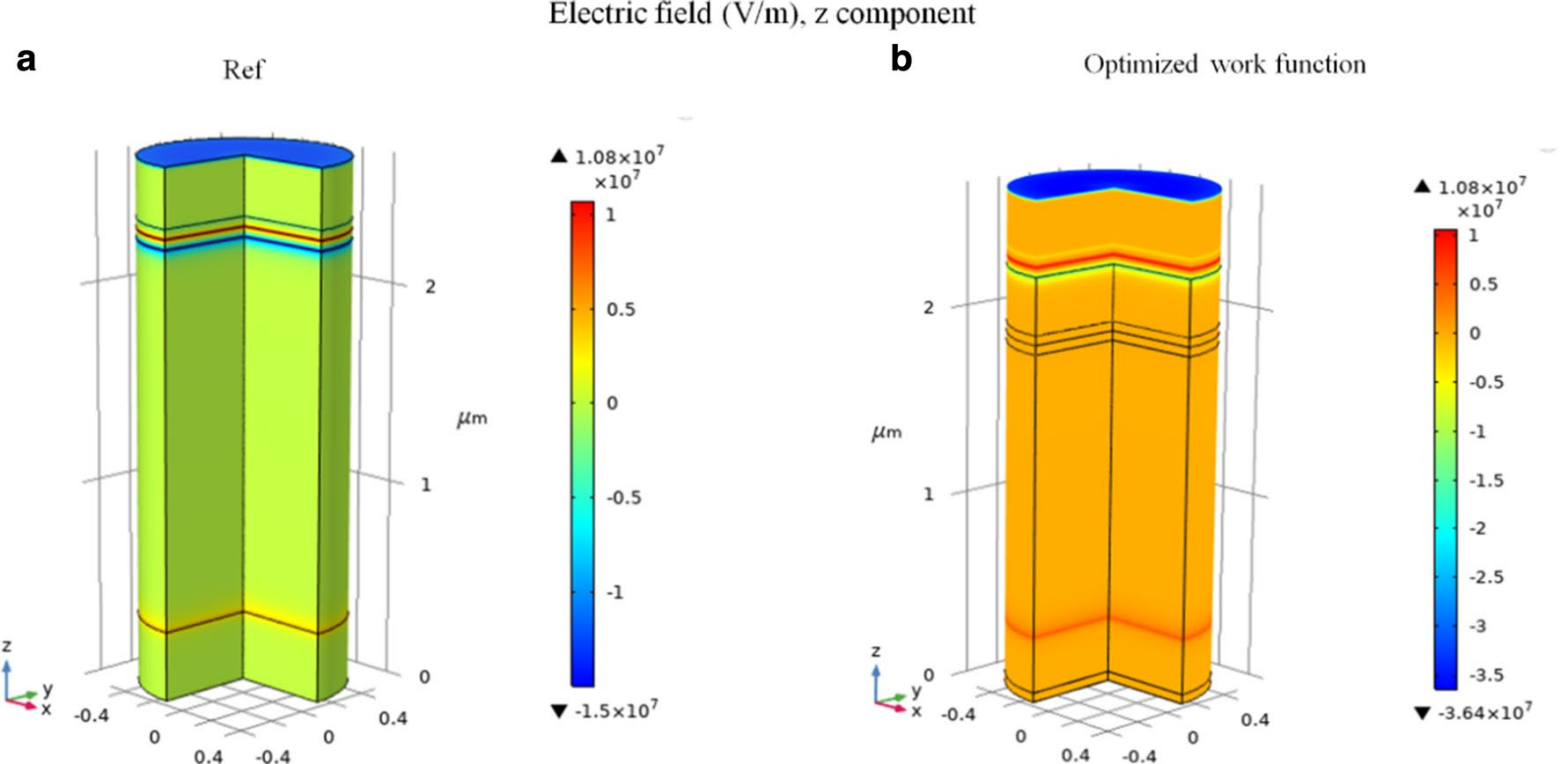
The electric field along the z component for (**a**) validated (Ref), and (**b**) simulated model (optimized work function).

Figure 3 indicates the effect of the potential barrier on performance of the simulated cell, which is derived by:

20 $${\varphi }_{B0}={W}_{F}-{\chi }_{SF}$$

where ${\varphi }_{B0}$ is the potential barrier in the front contact, ${W}_{F}$ is the metal work function of the front contact, and ${\chi }_{SF}$ is the electron affinity of the connected semiconductor layer to the front contact. According to Fig. 3, the lower the value of ${\varphi }_{B0}$, the higher the open-circuit voltage (V_OC_) and the short-circuit current density (J_SC_), so the result would be increased in the cell PCE. As one can see in Fig. 4, the better efficiency at ${\varphi }_{B0}=-0.3$ eV with work function of 4.1 eV is recorded.

Moreover, Fig. 4 depicts the comparison between the electric field (along z component) of the validated and simulated case, after optimization of the work function. As one can notice, the electric field has been improved from − 1.50 × 10^7^ to − 3.64 × 10^7^ (V/m), and this leads to higher open-circuit voltage (V_OC_) and short-circuit current density (J_SC_), as well as the device performance.

### Investigation of the cell performance based on the different possible defects and varying defect densities

The simulated results are based on the existence of possible defects and their energy level. Table 3 shows the defect position of (a) CZTS, and (b) CZTSe. Since CZTSSe absorber layer is considered based on the S/(S + Se) ratio, all types of defects could be possible for this layer. (+) and (−) denote donor and acceptor defects with their charges respectively.

**Table 3:** The energy level position of acceptor and donor lattice defects (inside the bandgap with respect to the VBM positions) for CZTS (left column), and CZTSe (right column) layers based on the Density Functional Theory (DFT) calculations^36^.

CZTS		CZTSe	
Defect	E_defect_ (meV)	Defect	E_defect_ (meV)
V_Cu_ (−/0)	30	V_Cu_ (−/0)	40
V_Zn_ (2−/−)	260	V_Zn_ (2−/−)	230
V_Zn_ (−/0)	120	V_Zn_ (−/0)	130
V_Sn_ (4−/3−)	900	V_Sn_ (4−/3−)	550
V_Sn_ (3−/2−)	600	V_Sn_ (3−/2−)	400
V_Sn_ (2−/−)	370	V_Sn_ (2−/−)	260
V_Sn_ (−/0)	180	V_Sn_ (−/0)	170
Cu_Zn_ (−/0)	146	Cu_Zn_ (−/0)	105
Cu_Sn_ (3−/2−)	590	Cu_Sn_ (3−/2−)	440
Cu_Sn_ (2−/−)	440	Cu_Sn_ (2−/−)	300
Cu_Sn_ (−/0)	210	Cu_Sn_ (−/0)	170
Zn_Sn_ (2−/−)	280	Zn_Sn_ (2−/−)	170
Zn_Sn_ (−/0)	140	Zn_Sn_ (−/0)	90
Zn_Cu_ (0/+)	1390	Zn_Cu_ (0/+)	920
Sn_Cu_ (0/+)	1520	Sn_Cu_ (0/+)	1100
Sn_Cu_ (+/3+)	380	Sn_Cu_ (+/3+)	430
Sn_Zn_ (0/+)	500	Sn_Zn_ (0/+)	670
Sn_Zn_ (+/2+)	500	Sn_Zn_ (+/2+)	550
Cu_i_ (0/+)	1400	Cu_i_ (0/+)	940
Zn_i_ (0/+)	1020	Zn_i_ (0/+)	700
Zn_i_ (+/2+)	870	Zn_i_ (+/2+)	680
V_S_ (0/2+)	760	V_Se_ (0/2+)	350

**Figure 5:**
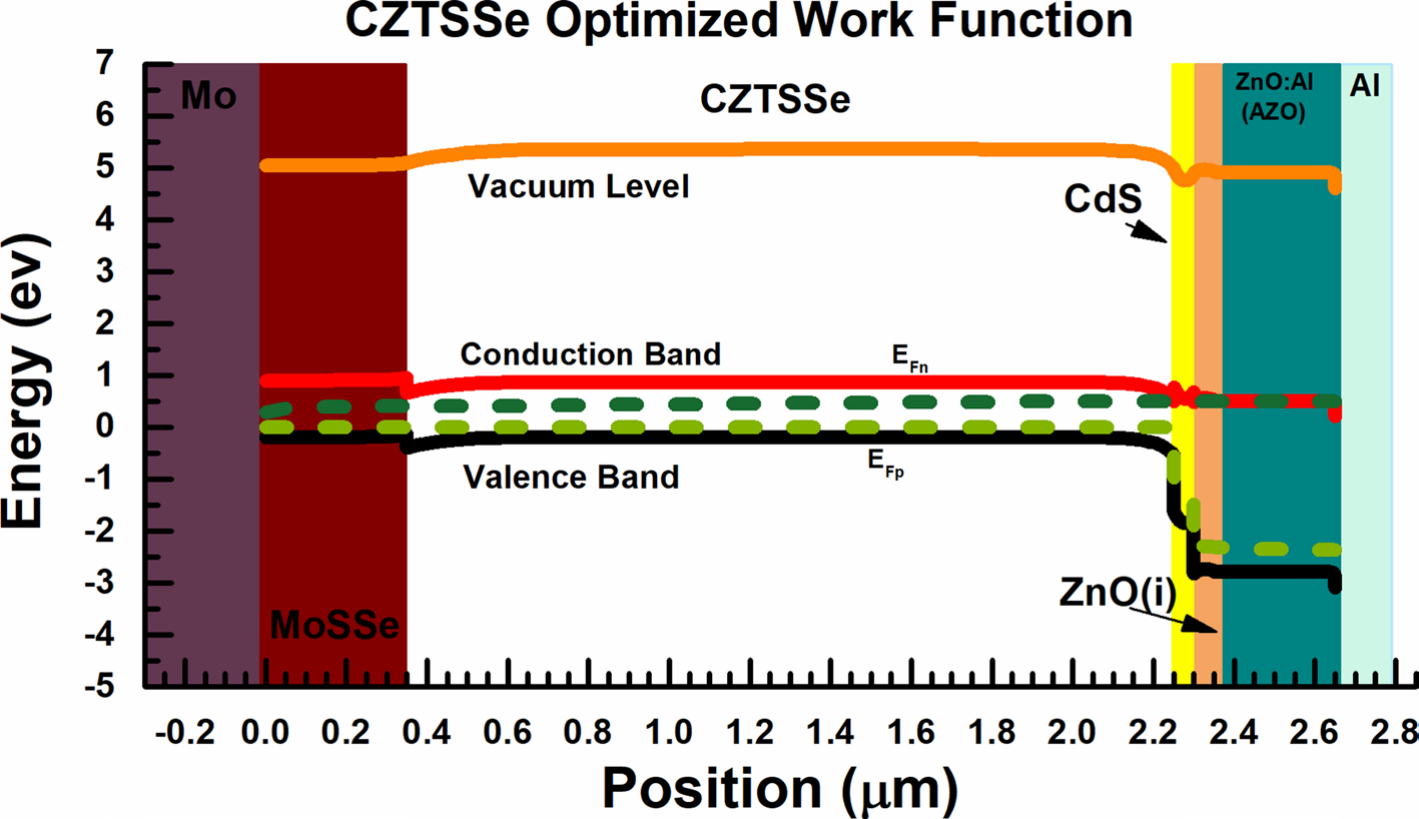
Band diagram of CZTSSe solar cell with optimized value of the work function.

Moreover, Fig. 5 represents the band diagram of the CZTSSe-based solar cell under the standard source of illumination AM 1.5 (at the V_OC_ voltage). According to Fig. 5, by optimizing the value of work function, the conduction and valence band have been changed, and this enhances the carrier transfer from absorber layer to back contact, so an increment in open-circuit voltage (V_OC_) and the cell efficiency has been recorded.

### Optimization of defect energy and density

**Figure 6:**
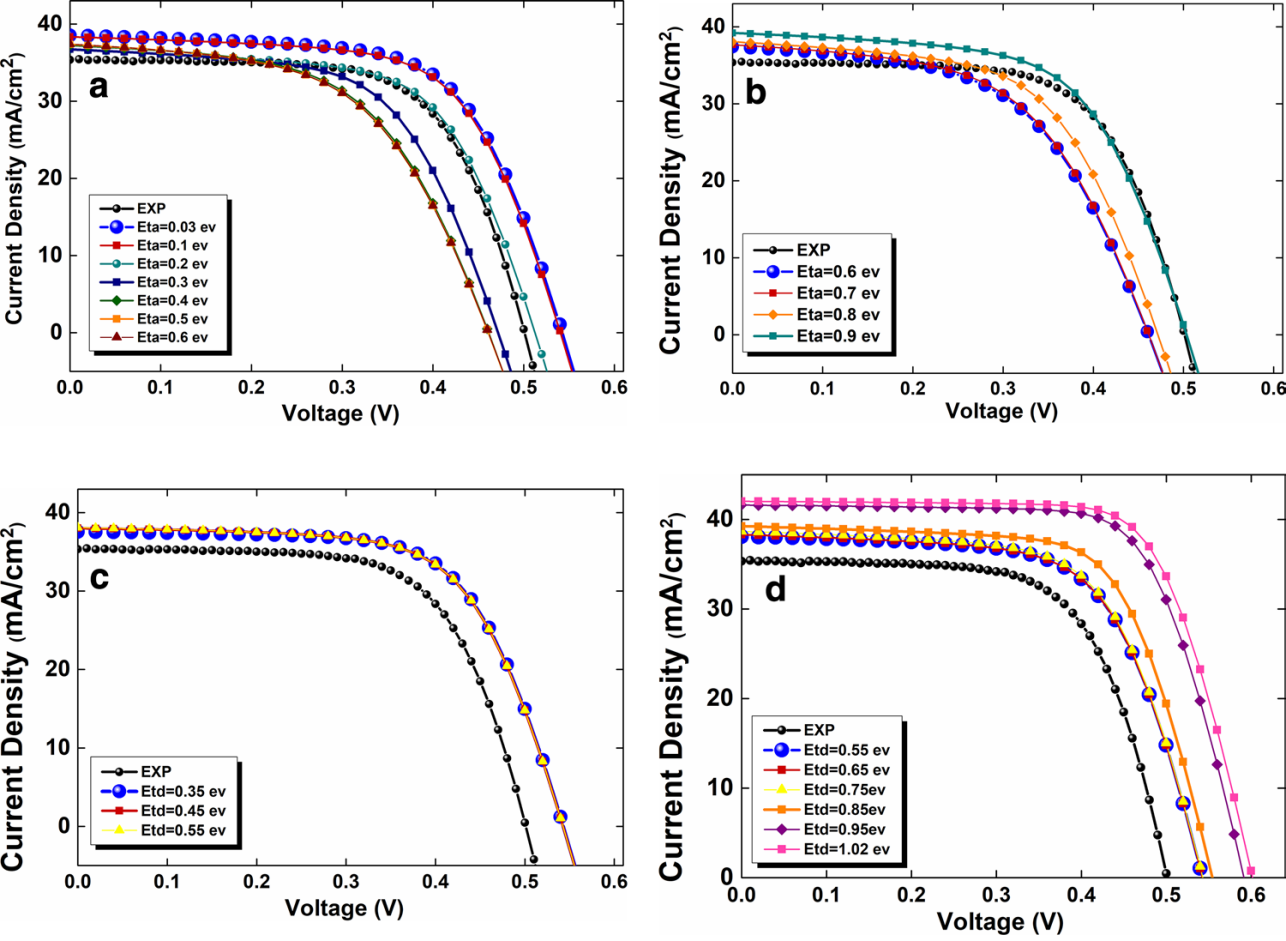
The effect of the absorber bulk defects positions on the current density versus voltage in range of (**a**) 0.03 eV to 0.6 eV (acceptor defect), (**b**) 0.6 eV to 0.9 eV (acceptor defect), (**c**) 0.35 eV to 0.55 eV (donor defect), and (**d**) 0.55 eV to 1.02 eV (donor defect) (the choice of ranges was based on Density Functional Theory (DFT) calculations^36^).

The value of 1.02 eV is the maximum value that the bandgap could attain as illustrated in Table 3. So, we ought to choose defects energies less than or equal to this value. The average value of the electron Fermi level (E_fn_) energy is 0.6 eV above the Valence Band Maximum (VBM) of the absorber layer. The effect of acceptor defects in the absorber layer [in the range of (1) 0.03 eV to 0.6 eV (Fig. 6a), and (2) 0.6 eV to 0.9 eV (Fig. 6b)] on the current density versus voltage (J-V) has been investigated. As indicated in Fig. 6a, acceptor defects with energy of ${E}_{t}=0.03$ eV record higher V_OC_ and J_sc_, which improve the cell PCE. Therefore, ${E}_{t,a}=0.03$ eV is the optimum energy value for the acceptor defect, and we used that as a defect energy value for simulating our device to find out the optimum value for donor defect energy. Moreover, the investigation of donor defects positions has been done in range of (1) 0.35 eV to 0.55 eV (Fig. 6c), and (2) 0.55 eV to 1.02 eV (Fig. 6d). Consequently, we recorded an increment in open-circuit voltage (V_OC_) and Jsc when the donor defect energy is ${E}_{t,d}=1.02$ eV. In a nutshell, we were able to claim that ${E}_{t,a}=0.03$ eV and ${E}_{t,d}=1.02$ eV are optimum energy values for acceptor and donor defects, respectively.

**Figure 7:**
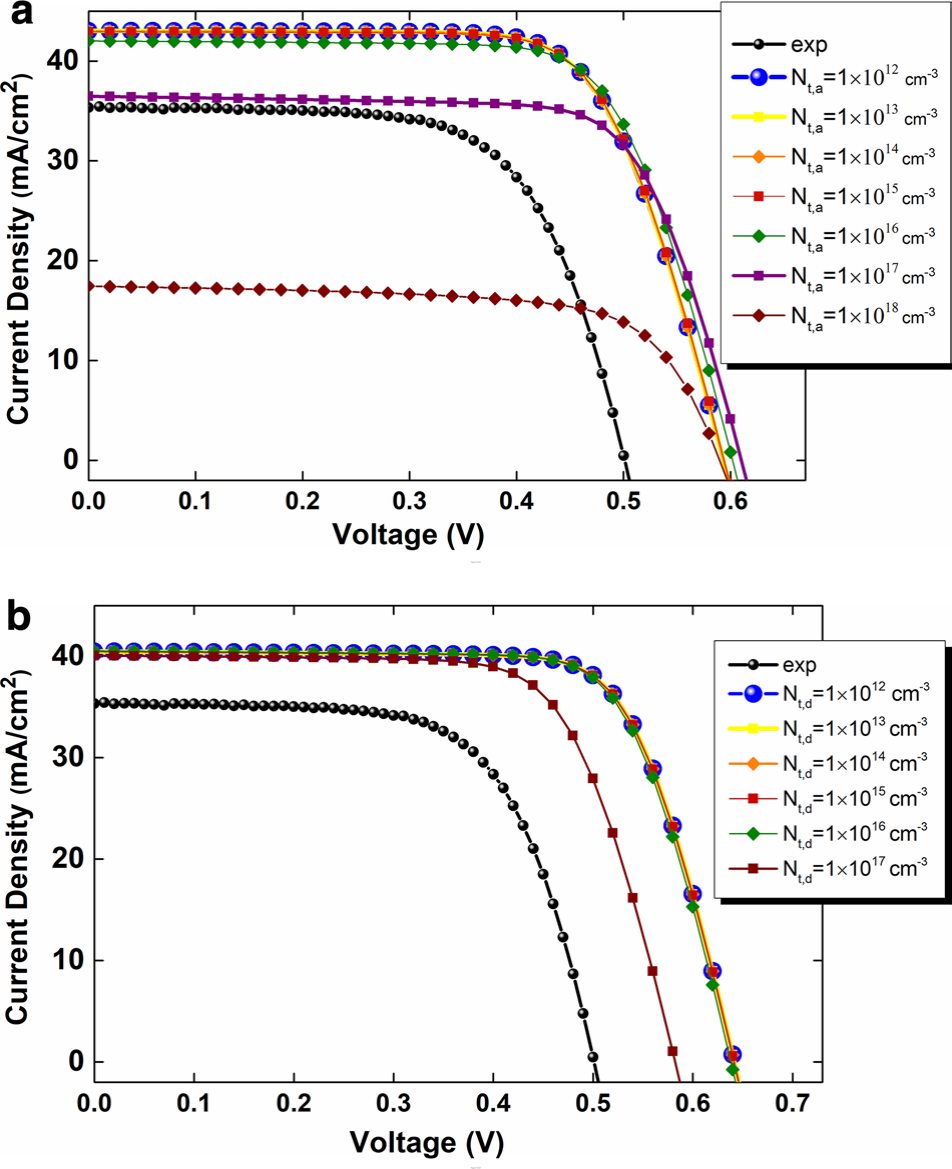
The effect of the absorber bulk defects position on the Current density versus voltage curves in range of (**a**) 1.0 × 10^12^ cm^−3^ to 1.0 × 10^18^ cm^−3^ (acceptor defect density), and (**b**) 1.0 × 10^12^ cm^−3^ to 1.0 × 10^17^ cm^−3^ (donor defect density) (the choice of ranges was based on Density Functional Theory (DFT) calculations^36^).

As the next step, to find the optimum density value, acceptor densities in the range of 1.0 × 10^12^ cm^−3^ to 1.0 × 10^18^ cm^−3^ and donor defects densities in the range of 1.0 × 10^12^ cm^−3^ to 1.0 × 10^17^ cm^−3^ have been investigated. As seen in Fig. 7a, ${N}_{t, a}={10}^{12}$ cm^−3^ (acceptor defect density) with higher short-circuit current density (J_sc_), and open-circuit voltage (V_OC_) is the best density value for the acceptor density. Hence, we have simulated the device with ${N}_{t,a}={10}^{12}$ cm^−3^ as an optimum defects density value to find the optimum density for donor defects. So, based on our calculations, ${N}_{t, d}={10}^{12}$ cm^−3^ (donor defect density) is the optimum carrier density of donor defects (Fig. 7b). Subsequently, we can assert that ${N}_{t, a}={N}_{t, d}={10}^{12}$ cm^−3^ are optimum parameters for acceptor and donor defects. Thus, by having optimized values of energies and carrier densities of defects, we are able to simulate a CZTSSe solar cell with higher efficiency (19.06%) than previous works. Also, in Table 4 we present harmful and benign defects for the device performance. As a critical point, closer defects to the electron Fermi level (E_fn_) position or ~ *Eg*/2 (related equations are available in Part S2 in supplementary) cause the absorber electrical conductivity deterioration, which increases the recombination rate, and reduces the open-circuit voltage (V_OC_), short-circuit current density (J_sc_) and PCE. Also, the increment of acceptor and donor defect densities increases the possibility of electron-trapping in the bandgap, and this leads to a reduction in the absorber electrical conductivity and PCE.

**Table 4:** Harmful and benign defects based on their influence on the cell performance.

Defects		Approximate E_t_ (eV)	Effect on the cell performance in ${N}_{t}<{10}^{16}$
in CZTS	in CZTSe		
V_Cu_ (−/0)	V_Cu_ (−/0)	0.030	Benign
V_Zn_ (−/0), Cu_Zn_ (−/0), Zn_Sn_ (−/0)	V_Zn_ (−/0), Cu_Zn_ (−/0), Zn_Sn_ (−/0)	0.10	Benign
V_Sn_ (−/0), Cu_Sn_ (−/0)	V_Zn_ ((2−/−), Cu_Zn_ (−/0), Zn_Sn_ (2−/−)	0.20	Benign
V_Zn_ (2−/−), Zn_Sn_ (2−/−)	V_Sn_ (2−/−), Cu_Sn_ (2−/−)	0.30	Harmful
V_Sn_ (2−/−)	V_Se_ (0/2+)	0.35	Harmful
Sn_Cu_ (+/3+)	V_Sn_ (3−/2−)	0.40	Harmful
Cu_Sn_ (2−/−)	Cu_Sn_ (3−/2−), Sn_Cu_ (+/3+)	0.45	Harmful
Sn_Zn_ (0/+), Sn_Zn_ (+/2+)	–	0.50	Harmful
–	V_Sn_ (4−/3−), Sn_Zn_ (+/2+)	0.55	Harmful
V_Sn_ (3−/2−), Cu_Sn_ (3−/2−)	–	0.60	Harmful
–	Sn_Zn_ (0/+)	0.65	Harmful
–	Zn_i_ (0/+), Zn_i_ (+/2+)	0.70	Harmful
V_S_ (0/2+)	–	0.75	Harmful
–	–	0.80	Harmful
Zn_i_ (+/2+)	–	0.85	Harmful
V_Sn_ (4−/3−), Zn_i_ (+/2+)	Zn_Cu_ (0/+)	0.90	Benign
–	Cu_i_ (0/+)	0.95	Benign
Zn_i_ (0/+)	Sn_Cu_ (0/+)	1.020	Benign

**Figure 8:**
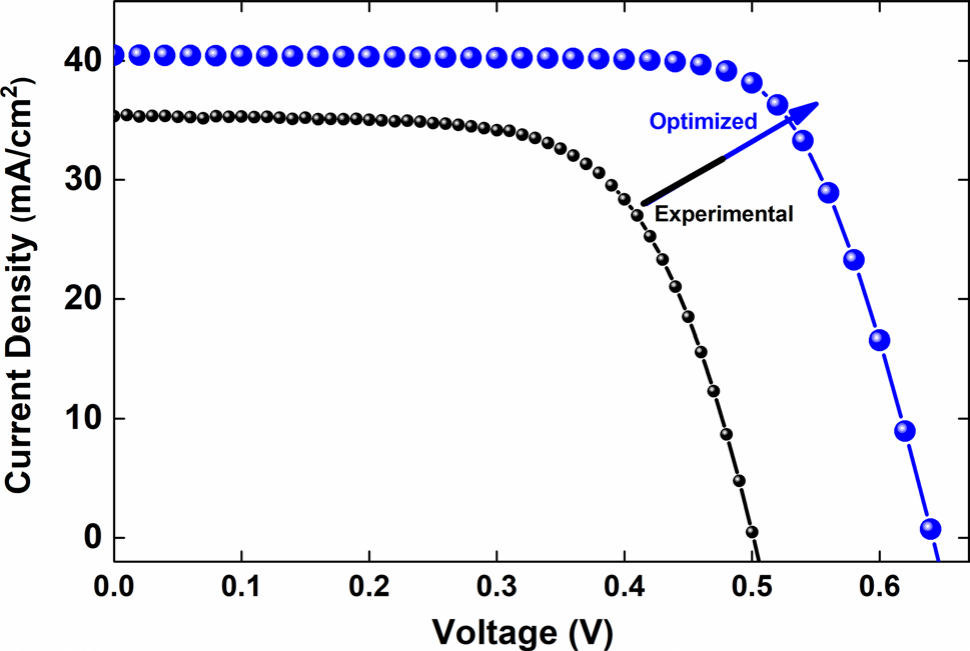
Optimized J–V characteristics of the cell in comparison with the experimental data.

Figure 8 represents the final optimized J–V characteristics of the cell in comparison with the experimental data. Obviously, this comparison indicates that the simulation results are in good agreement with experimental data, as it shows an impressive increment in the cell performance.

### Investigation of the device performance according to the recombination rate distribution

**Figure 9:**
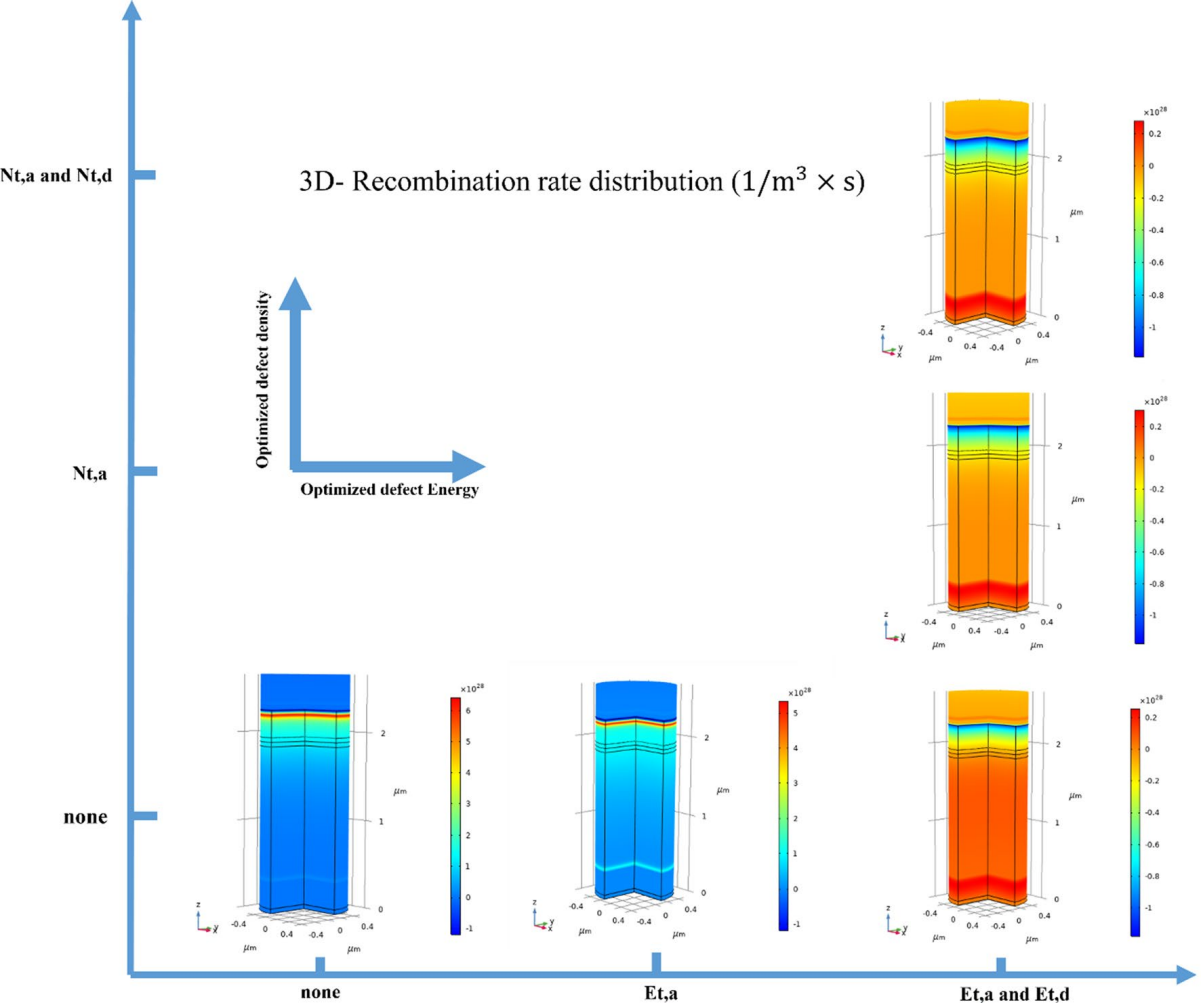
The recombination rate of distribution (1/m^3^ × s) for different cases (optimized Eta,d, Nta,d and non-optimized).

It is a crucial point to reduce recombination rate for enhancing the cell performance. So, Fig. 9 illustrates the optimized defect energy and density values along the z-component. As shown in Fig. 9, we introduce a 2-D coordinate system, where the x-component depicts optimized defect energy, and the y-component demonstrates optimized defect density. The ($$None)$$ value shows the recombination rate without the optimization of the defect energy and the defect density. Optimizing the acceptor defect energy ($${E}_{t,a}$$) with non-optimized acceptor defect density ($${N}_{t,a}$$), causes lower recombination rate than the previous part in ($$None)$$ mode. The next step for reducing the recombination rate is optimizing $${E}_{t,a} and {E}_{t,d}$$ without the optimization of the $${N}_{t,a}$$. Also, optimization of $${E}_{t,a} and {E}_{t,d}$$ with $${N}_{t,a}$$ reduces the recombination rate, but the best device performance has been recorded when we optimized the $${E}_{t,a} and {E}_{t,d}$$, and the $${N}_{t,a} and {N}_{t,d}$$, which shows a dramatic reduction in the recombination rate with the applied voltage of 0.9 V.

### Characterization harmful and benign defects (based on their effects on the cell efficiency)

All of the defects have been formed in the CZTSSe layer are not harmful, and there are benign defects in the CZTSSe absorber layer, and recognition of harmful or benign defects is based on their influence on the cell performance. Benign defects reduce carrier recombination on the grain boundary by their segregation. For example, Zn_Sn_ (as a benign defect) provides deep levels in the CZTSSe absorber by breaking or weakening bonds, and also create barrier for holes and easy transport of electrons through the grain boundaries^30,72,73^. Our evaluation for harmful and benign defects was based on the investigation of available defects in CZTS and CZTSe, and their energy E_t_ (eV) with respect to the Valence Band Maximum (VBM) of the absorber layer. According to the Table 4, benign defects have no negative effect on the cell performance, in contradict, harmful defects cause the cell PCE deterioration. As a crucial point, defects with density (${N}_{t}$) higher than 10^16^ (1/cm^3^) are harmful for the cell efficiency, even if they are far from the electron Fermi level (E_fn_) energy. It should be noted that, defects with the position higher than the Conduction Band Minimum (CBM) are not considered because they are meaningless (Zn_Cu_ (0/+), Sn_Cu_ (0/+), Cu_i_ (0/+)).

## Conclusion

Controlling the defects and secondary phases of CZTSSe absorber layer have significant influence on the in kesterite solar cell performance improvement. Previous studies have demonstrated that, synthesis of CZTSSe:Na nanocrystals and controlling the S/(S + Se), Cu/(Zn + Sn), and Zn/Sn ratios (Stoichiometry) have a significant effect on the reduction of trap-assisted recombination (Shockley–Read–Hall recombination model). Moreover, adding Graphene and Graphyne to the absorber precursors in order to enhance the conductivity might also improve the total mobility and reduces the recombination. In this paper, the CZTSSe absorber materials in kesterite solar cell by using the Finite Element Method (FEM) with (1) electrical, and (2) optical approaches has been investigated and simulation outcomes have been validated by the experimental data. We first optimized the potential barrier effect in the front contact and the connected semiconductor layer. It is found that the lower the value of ${\varphi }_{B0}=-0.3$ eV leads the higher the open-circuit voltage (V_OC_) and PCE. In the next step, a screening-based approach has been employed to study the cell efficiency over a wide range of defect densities. Two categorized defect types including benign defects (${N}_{t}<{10}^{16}$ cm^−3^, N_t_ defines trap density) and harmful defects ${(N}_{t}>{10}^{16}$ cm^−3^) in the absorber bandgap in the CZTSSe solar cell, by analyzing their position changes with respect to the electron Fermi level (E_fn_) and the Valence Band Maximum (VBM) positions have been recognized. The optimum values of ${E}_{t, a}$ = 0.03 eV, ${E}_{t, d}\hspace{0.17em}$= 1.02 eV and ${N}_{t, a}={N}_{t, d}={10}^{12}$ cm^−3^ for accepter and donor have been obtained, respectively. Optimization of potential barrier effect in front contact (${\varphi }_{B0}$), classifying the harmful and benign defects, and reduction in defects densities helps us to record the PCE of 19.06%, which is a remarkable increment in comparison with other results and makes the CZTSSe solar cells as a promising candidate for industrial and commercial applications.

## Methods

The precursor metal films have been deposited on a Soda Lime Glass (SLG) substrate, which has been coated with a 600 nm-thick Mo. Zn and Sn layers have been consecutively deposited by co-sputtering with the Cu layer. It should be noted that a single layer of Cu was finally deposited to control the Cu composition. Cu/Cu–Sn/Cu–Zn/Mo/Glass is the stack structure of the deposited precursor. The layers were deposited under sputtering powers of 150 W, 300 W, and 300 W for the Cu, Zn and Sn targets, respectively, at a working pressure of 1 mTorr at Ar atmosphere. It is worth mentioning that, the metal precursors were reacted and annealed in a furnace. The CZTSSe absorption layer was synthesized through a sulfo-selenization process and heat-treated in a Se Shot and Ar/H2S gas atmosphere. Also, 480 °C was the final temperature of the heat treatment, and lasted for 10 min. Cu2ZnSn(S,Se)_4_ (CZTSSe) absorber layer was coated with a 50 nm CdS buffer layer by chemical bath deposition to fabricate the solar cell,. Afterward, a 50 nm intrinsic ZnO (ZnO(i)) layer and a 300 nm Al-doped ZnO (AZO (ZnO:Al)) layer were deposited by RF sputtering. At last, a 2 µm Al grid, and a 110 nm MgF_2_ were deposited by the e-beam evaporator. It should be noted that, the MgF_2_ layer was deposited as an antireflection coating layer. Figure S1 in the supporting file illustrates the cross section FESEM image of the cell. It should be noted that, the substrate temperature and deposition time play a crucial role in determining the quality and composition of kesterite films. The current–voltage characteristics were measured under a simulated air mass 1.5 global (AM 1.5 G) spectrum in an illumination of 100 mW/cm^2^ (1 sun) using a solar simulator (Newport Co., model 94022A). The grading composition of S/S + Se ratio has been shown in Figure S3.

## Supplementary information

Supplementary Information.

## Data Availability

The data that support the findings of this study are available from the corresponding author (M.M. and E.Y.), upon reasonable request.

## References

[1] Müller J, Rech B, Springer J, Vanecek M (2004). TCO and light trapping in silicon thin film solar cells. Sol. Energy.

[2] Wallace SK, Mitzi DB, Walsh A (2017). The steady rise of kesterite solar cells. ACS Energy Lett..

[3] Polman A, Knight M, Garnett EC, Ehrler B, Sinke WC (2016). Photovoltaic materials: Present efficiencies and future challenges. Science.

[4] Tao J (2019). Solution-processed SnO_2_ interfacial layer for highly efficient Sb_2_Se_3_ thin film solar cells. Nano Energy.

[5] Asim N (2012). A review on the role of materials science in solar cells. Renew. Sustain. Energy Rev..

[6] Minbashi M, Ghobadi A, Ehsani MH, Rezagholipour Dizaji H, Memarian N (2018). Simulation of high efficiency SnS-based solar cells with SCAPS. Sol. Energy.

[7] Otoufi MK (2020). Enhanced performance of planar perovskite solar cells using TiO_2_/SnO_2_ and TiO_2_/WO_3_ bilayer structures: Roles of the interfacial layers. Sol. Energy.

[8] Cui Y (2019). Over 16% efficiency organic photovoltaic cells enabled by a chlorinated acceptor with increased open-circuit voltages. Nat. Commun..

[9] Ge J (2017). Oxygenated CdS buffer layers enabling high open-circuit voltages in earth-abundant Cu_2_BaSnS_4_ thin-film solar cells. Adv. Energy Mater..

[10] Ghobadi A (2020). Simulating the effect of adding BSF layers on Cu_2_BaSnSSe_3_ thin film solar cells. Opt. Mater. (Amst).

[11] Jackson P (2016). Effects of heavy alkali elements in Cu(In,Ga)Se_2_ solar cells with efficiencies up to 22.6%. Phys status solidi (RRL)-Rapid Res. Lett..

[12] Green MA (2019). Solar cell efficiency tables (version 54). Prog. Photovolt. Res. Appl..

[13] Poplawsky JD (2016). Structural and compositional dependence of the CdTe_x_Se_1–x_ alloy layer photoactivity in CdTe-based solar cells. Nat. Commun..

[14] Katagiri H (1997). Preparation and evaluation of Cu_2_ZnSnS_4_ thin films by sulfurization of E–B evaporated precursors. Sol. Energy Mater. Sol. Cells.

[15] Wang W (2014). Device characteristics of CZTSSe thin-film solar cells with 12.6% efficiency. Adv. Energy Mater..

[16] Liu X (2016). The current status and future prospects of kesterite solar cells: A brief review. Prog. Photovolt. Res. Appl..

[17] Kim S (2018). Limiting effects of conduction band offset and defect states on high efficiency CZTSSe solar cell. Nano Energy.

[18] Katagiri H (2009). Development of CZTS-based thin film solar cells. Thin Solid Films.

[19] Walsh A, Chen S, Wei S, Gong X (2012). Kesterite thin-film solar cells: Advances in materials modelling of Cu_2_ZnSnS_4_. Adv. Energy Mater..

[20] Yang K-J (2016). A band-gap-graded CZTSSe solar cell with 12.3% efficiency. J. Mater. Chem. A.

[21] Minbashi M, Omrani MK, Memarian N, Kim D-H (2017). Comparison of theoretical and experimental results for band-gap-graded CZTSSe solar cell. Curr. Appl. Phys..

[22] Hironiwa D, Murata M, Ashida N, Tang Z, Minemoto T (2014). Simulation of optimum band-gap grading profile of Cu_2_ZnSn(S,Se)_4_ solar cells with different optical and defect properties. Jpn. J. Appl. Phys..

[23] Omrani MK, Minbashi M, Memarian N, Kim DH (2018). Improve the performance of CZTSSe solar cells by applying a SnS BSF layer. Solid State Electron..

[24] Todorov TK (2013). Beyond 11% efficiency: Characteristics of state-of-the-art Cu2ZnSn(S,Se)_4_ solar cells. Adv. Energy Mater..

[25] Son D-H (2019). Effect of solid-H_2_S gas reactions on CZTSSe thin film growth and photovoltaic properties of a 12.62% efficiency device. J. Mater. Chem. A.

[26] Bourdais S (2016). Is the Cu/Zn disorder the main culprit for the voltage deficit in kesterite solar cells?. Adv. Energy Mater..

[27] Chen S, Yang J-H, Gong X-G, Walsh A, Wei S-H (2010). Intrinsic point defects and complexes in the quaternary kesterite semiconductor Cu_2_ZnSnS_4_. Phys. Rev. B.

[28] Zhai Y-T (2011). Structural diversity and electronic properties of Cu_2_SnX_3_ (X= S, Se): A first-principles investigation. Phys. Rev. B.

[29] Giraldo S (2019). Progress and perspectives of thin film kesterite photovoltaic technology: A critical review. Adv. Mater..

[30] Kumar M, Dubey A, Adhikari N, Venkatesan S, Qiao Q (2015). Strategic review of secondary phases, defects and defect-complexes in kesterite CZTS–Se solar cells. Energy Environ. Sci..

[31] Chen S, Gong XG, Walsh A, Wei SH (2010). Defect physics of the kesterite thin-film solar cell absorber Cu_2_ZnSnS_4_. Appl. Phys. Lett..

[32] Haghighi M, Taghavinia N, Kim D-H, Mahdavi SM, Kordbacheh AA, Minbashi M (2018). A modeling study on utilizing SnS_2_ as the buffer layer of CZT(S,Se) solar cells. Sol. Energy.

[33] Barkhouse DAR, Gunawan O, Gokmen T, Todorov TK, Mitzi DB (2012). Device characteristics of a 10.1% hydrazine-processed Cu_2_ZnSn(Se,S)_4_ solar cell. Prog. Photovolt. Res. Appl..

[34] Shin D, Saparov B, Mitzi DB (2017). Defect engineering in multinary earth-abundant chalcogenide photovoltaic materials. Adv. Energy Mater..

[35] Chen S, Wang L-W, Walsh A, Gong XG, Wei S-H (2012). Abundance of CuZn + SnZn and 2CuZn + SnZn defect clusters in kesterite solar cells. Appl. Phys. Lett..

[36] Chen S, Walsh A, Gong X, Wei S (2013). Classification of lattice defects in the kesterite Cu_2_ZnSnS_4_ and Cu_2_ZnSnSe_4_ earth-abundant solar cell absorbers. Adv. Mater..

[37] Su Z (2015). Cation substitution of solution-processed Cu_2_ZnSnS_4_ thin film solar cell with over 9% efficiency. Adv. Energy Mater..

[38] Mutter D, Dunham ST (2015). Calculation of defect concentrations and phase stability in Cu_2_ZnSnS_4_ and Cu_2_ ZnSnSe_4_ from stoichiometry. IEEE J. Photovolt..

[39] Zhang Y (2012). Structural properties and quasiparticle band structures of Cu-based quaternary semiconductors for photovoltaic applications. J. Appl. Phys..

[40] Sun R (2017). High-sulfur Cu_2_ZnSn(S,Se)_4_ films by sulfurizing as-deposited CZTSe film: The evolutions of phase, crystallinity and S/(S + Se) ratio. J. Alloys Compd..

[41] Franckevičius M (2019). Efficiency improvement of superstrate CZTSSe solar cells processed by spray pyrolysis approach. Sol. Energy.

[42] Han J, Shin SW, Gang MG, Kim JH, Lee JY (2013). Crystallization behaviour of co-sputtered Cu_2_ZnSnS_4_ precursor prepared by sequential sulfurization processes. Nanotechnology.

[43] Hergert F, Hock R (2007). Predicted formation reactions for the solid-state syntheses of the semiconductor materials Cu_2_SnX_3_ and Cu_2_ZnSnX_4_ (X= S, Se) starting from binary chalcogenides. Thin Solid Films.

[44] Erkan ME, Chawla V, Repins I, Scarpulla MA (2015). Interplay between surface preparation and device performance in CZTSSe solar cells: Effects of KCN and NH_4_OH etching. Sol. Energy Mater. Sol. Cells.

[45] Xie H (2014). Impact of Sn(S,Se) secondary phases in Cu_2_ZnSn(S,Se)_4_ solar cells: A chemical route for their selective removal and absorber surface passivation. ACS Appl. Mater. Interfaces.

[46] Kim S-Y (2019). Void and secondary phase formation mechanisms of CZTSSe using Sn/Cu/Zn/Mo stacked elemental precursors. Nano Energy.

[47] Son D-H (2015). Growth and device characteristics of CZTSSe thin-film solar cells with 8.03 percent efficiency. Chem. Mater..

[48] Qi Y-F (2017). Engineering of interface band bending and defects elimination via a Ag-graded active layer for efficient (Cu,Ag)_2_ZnSn(S,Se)_4_ solar cells. Energy Environ. Sci..

[49] Hadke SH (2018). Synergistic effects of double cation substitution in solution-processed CZTS solar cells with over 10% efficiency. Adv. Energy Mater..

[50] Xiao Z, Meng W, Li JV, Yan Y (2017). Distant-atom mutation for better earth-abundant light absorbers: A case study of Cu_2_BaSnSe_4_. ACS Energy Lett..

[51] Shin D (2017). Earth-abundant chalcogenide photovoltaic devices with over 5% efficiency based on a Cu_2_BaSn(S,Se)_4_ absorber. Adv. Mater..

[52] Gershon T (2016). Photovoltaic materials and devices based on the alloyed kesterite absorber (Ag_x_Cu_1–x_)_2_ZnSnSe_4_. Adv. Energy Mater..

[53] Guchhait A (2016). Enhancement of open-circuit voltage of solution-processed Cu_2_ZnSnS_4_ solar cells with 7.2% efficiency by incorporation of silver. ACS Energy Lett..

[54] Khadka DB, Kim J (2015). Band gap engineering of alloyed Cu_2_ZnGe_x_Sn_1–x_Q4 (Q= S, Se) films for solar cell. J. Phys. Chem. C.

[55] Hages CJ (2015). Improved performance of Ge-alloyed CZTGeSSe thin-film solar cells through control of elemental losses. Prog. Photovolt. Res. Appl..

[56] Lie S (2018). Reducing the interfacial defect density of CZTSSe solar cells by Mn substitution. J. Mater. Chem. A.

[57] Zhou H (2013). Rational defect passivation of Cu_2_ZnSn(S,Se)_4_ photovoltaics with solution-processed Cu_2_ZnSnS_4_: Na nanocrystals. J. Am. Chem. Soc..

[58] Yang K-J (2015). Effects of the compositional ratio distribution with sulfurization temperatures in the absorber layer on the defect and surface electrical characteristics of Cu_2_ZnSnS_4_ solar cells. Prog. Photovolt. Res. Appl..

[59] Das S, Sa K, Alam I, Mahanandia P (2018). Synthesis of CZTS QDs decorated reduced graphene oxide nanocomposite as possible absorber for solar cell. Mater. Lett..

[60] Anderson TH, Civiletti BJ, Monk PB, Lakhtakia A (2020). Coupled optoelectronic simulation and optimization of thin-film photovoltaic solar cells. J. Comput. Phys..

[61] Sze SM, Ng KK (2006). Physics of Semiconductor Devices.

[62] Swinehart DF (1962). The Beer–Lambert law. J. Chem. Educ..

[63] Markowich PA, Ringhofer CA, Schmeiser C (2012). Semiconductor Equations.

[64] Yousefi M, Minbashi M, Monfared Z, Memarian N, Hajjiah A (2020). Improving the efficiency of CZTSSe solar cells by engineering the lattice defects in the absorber layer. Sol. Energy.

[65] Shockley W, Read WT (1952). Statistics of the recombinations of holes and electrons. Phys. Rev..

[66] Timmo K (2010). Sulfur-containing Cu_2_ZnSnSe_4_ monograin powders for solar cells. Sol. Energy Mater. Sol. Cells.

[67] Guo Q (2010). Fabrication of 7.2% efficient CZTSSe solar cells using CZTS nanocrystals. J. Am. Chem. Soc..

[68] Redinger A, Berg DM, Dale PJ, Siebentritt S (2011). The consequences of kesterite equilibria for efficient solar cells. J. Am. Chem. Soc..

[69] Todorov T (2011). Progress towards marketable earth-abundant chalcogenide solar cells. Thin Solid Films.

[70] Todorov TK, Reuter KB, Mitzi DB (2010). High-efficiency solar cell with earth-abundant liquid-processed absorber. Adv. Mater..

[71] Mitzi DB, Gunawan O, Todorov TK, Wang K, Guha S (2011). The path towards a high-performance solution-processed kesterite solar cell. Sol. Energy Mater. Sol. Cells.

[72] Persson C, Zunger A (2003). Anomalous grain boundary physics in polycrystalline CuInSe_2_: The existence of a hole barrier. Phys. Rev. Lett..

[73] Yan Y (2007). Electrically benign behavior of grain boundaries in polycrystalline CuInSe_2_ films. Phys. Rev. Lett..

