# Supplementary material for: Efficiency enhancement of CZTSSe solar cells via screening the absorber layer by examining of different possible defects
Source: Sci Rep. 2020 Dec 11;10:21813. doi: 10.1038/s41598-020-75686-2 (PMC7733511; doi:10.1038/s41598-020-75686-2)
Supplement: Supplementary file 1 — Supplementary Information. [file 41598_2020_75686_MOESM1_ESM.pptx]

## Slide 1
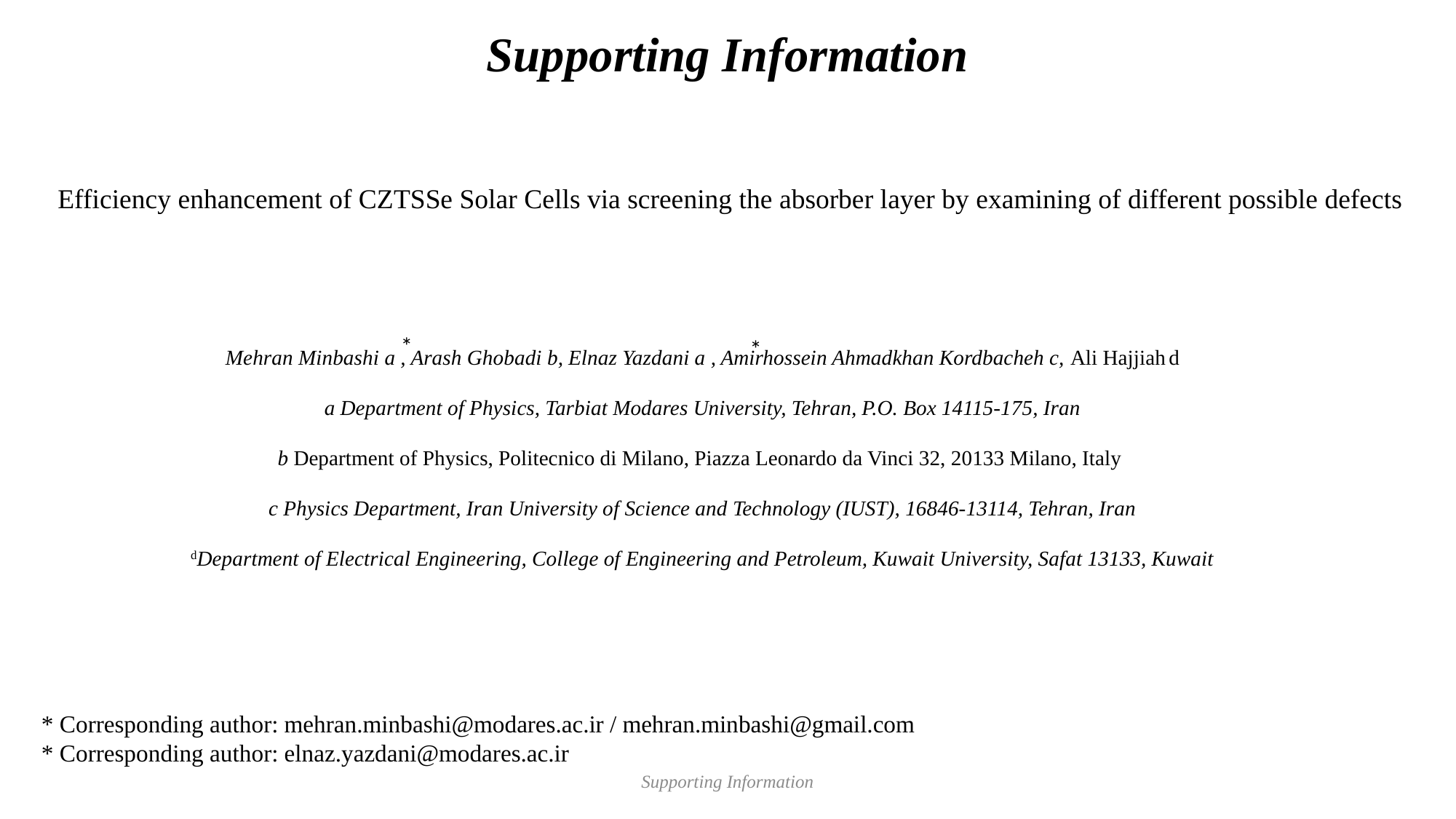

Supporting Information
Efficiency enhancement of CZTSSe Solar Cells via screening the absorber layer by examining of different possible defects
Mehran Minbashi a , Arash Ghobadi b, Elnaz Yazdani a , Amirhossein Ahmadkhan Kordbacheh c, Ali Hajjiah d
a Department of Physics, Tarbiat Modares University, Tehran, P.O. Box 14115-175, Iran
b Department of Physics, Politecnico di Milano, Piazza Leonardo da Vinci 32, 20133 Milano, Italy
c Physics Department, Iran University of Science and Technology (IUST), 16846-13114, Tehran, Iran
dDepartment of Electrical Engineering, College of Engineering and Petroleum, Kuwait University, Safat 13133, Kuwait
*
*
* Corresponding author: mehran.minbashi@modares.ac.ir / mehran.minbashi@gmail.com
* Corresponding author: elnaz.yazdani@modares.ac.ir
Supporting Information

## Slide 2
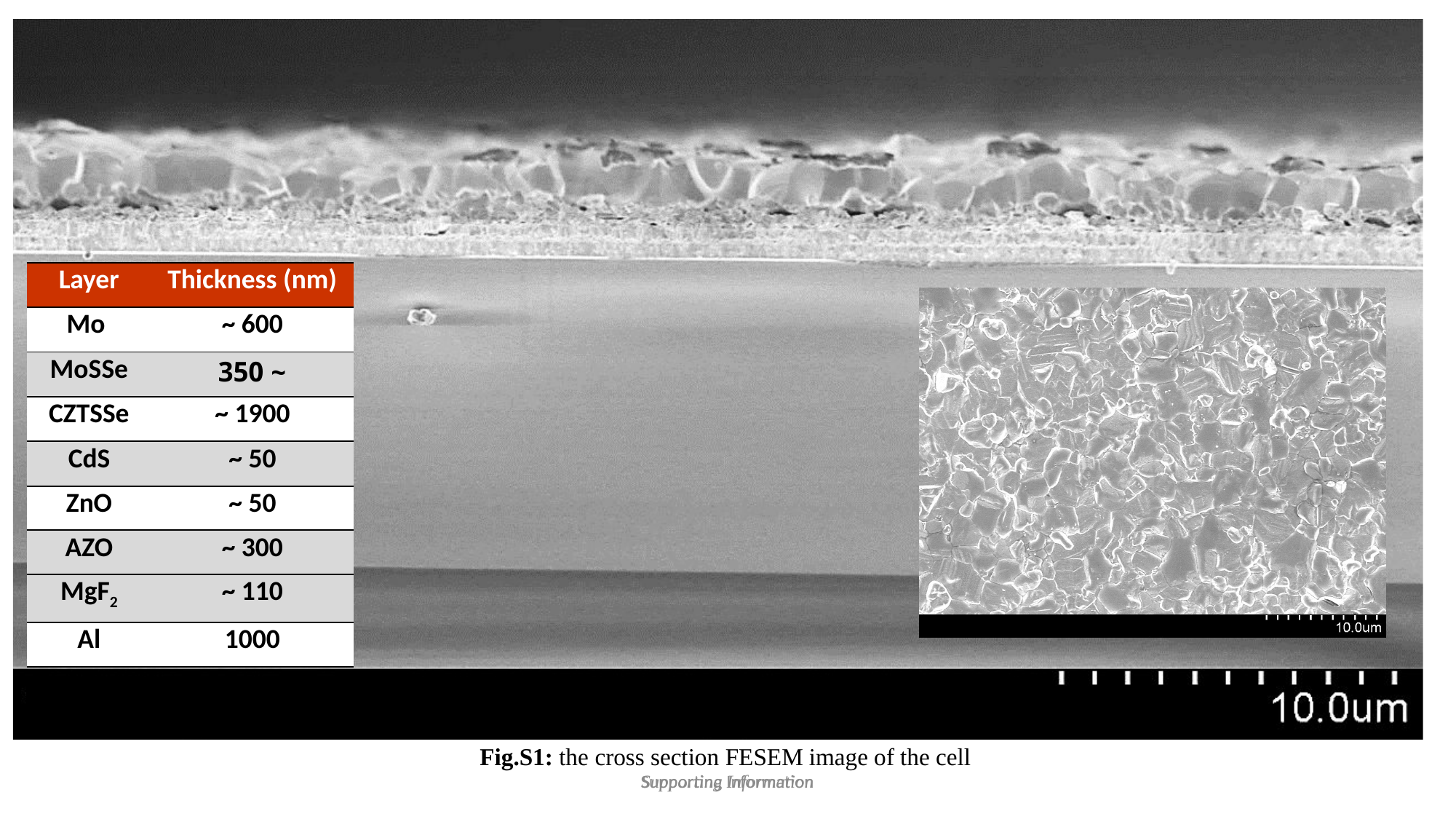

| Layer | Thickness (nm) |
| --- | --- |
| Mo | ~ 600 |
| MoSSe | ~ 350 |
| CZTSSe | ~ 1900 |
| CdS | ~ 50 |
| ZnO | ~ 50 |
| AZO | ~ 300 |
| MgF2 | ~ 110 |
| Al | 1000 |
Fig.S1: the cross section FESEM image of the cell
Supporting Information
Supporting Information

## Slide 3
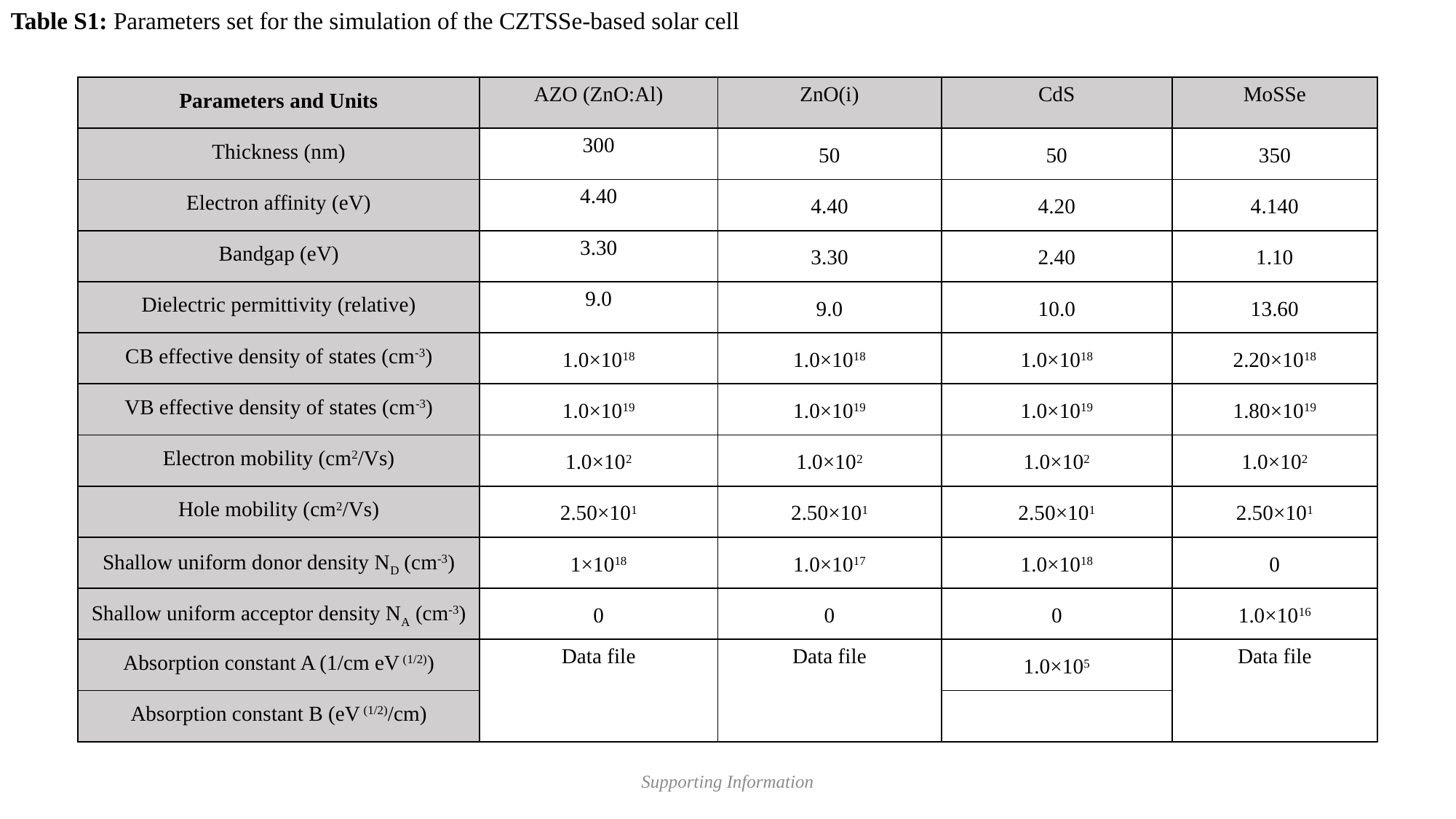

Table S1: Parameters set for the simulation of the CZTSSe-based solar cell
| Parameters and Units | AZO (ZnO:Al) | ZnO(i) | CdS | MoSSe |
| --- | --- | --- | --- | --- |
| Thickness (nm) | 300 | 50 | 50 | 350 |
| Electron affinity (eV) | 4.40 | 4.40 | 4.20 | 4.140 |
| Bandgap (eV) | 3.30 | 3.30 | 2.40 | 1.10 |
| Dielectric permittivity (relative) | 9.0 | 9.0 | 10.0 | 13.60 |
| CB effective density of states (cm-3) | 1.0×10^18^ | 1.0×10^18^ | 1.0×10^18^ | 2.20×10^18^ |
| VB effective density of states (cm-3) | 1.0×10^19^ | 1.0×10^19^ | 1.0×10^19^ | 1.80×10^19^ |
| Electron mobility (cm2/Vs) | 1.0×10^2^ | 1.0×10^2^ | 1.0×10^2^ | 1.0×10^2^ |
| Hole mobility (cm2/Vs) | 2.50×101 | 2.50×101 | 2.50×101 | 2.50×101 |
| Shallow uniform donor density ND (cm-3) | 1×10^18^ | 1.0×10^17^ | 1.0×10^18^ | 0 |
| Shallow uniform acceptor density NA (cm-3) | 0 | 0 | 0 | 1.0×10^16^ |
| Absorption constant A (1/cm eV (1/2)) | Data file | Data file | 1.0×10^5^ | Data file |
| Absorption constant B (eV (1/2)/cm) | | | | |
Supporting Information

## Slide 4
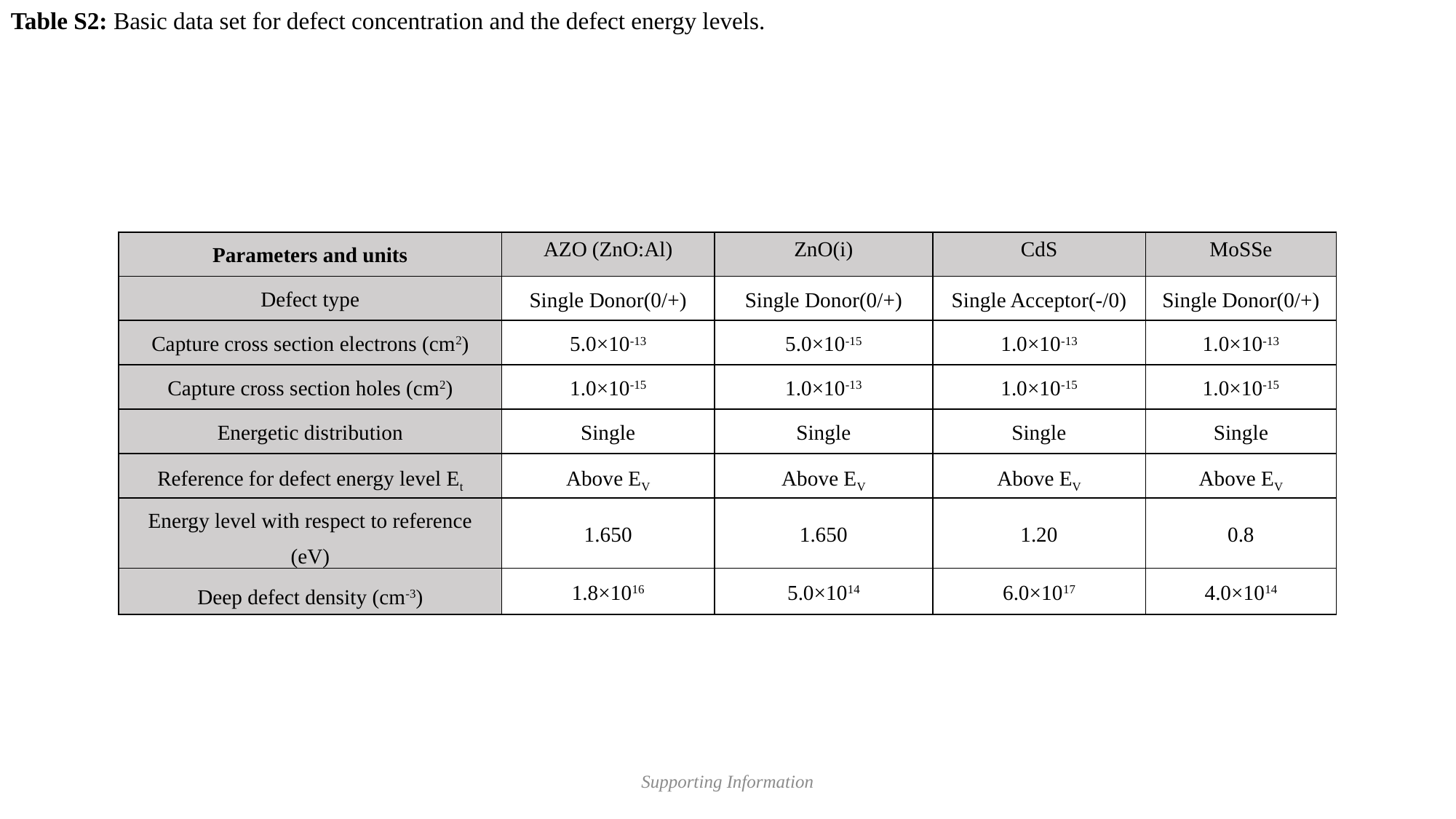

Table S2: Basic data set for defect concentration and the defect energy levels.
| Parameters and units | AZO (ZnO:Al) | ZnO(i) | CdS | MoSSe |
| --- | --- | --- | --- | --- |
| Defect type | Single Donor(0/+) | Single Donor(0/+) | Single Acceptor(-/0) | Single Donor(0/+) |
| Capture cross section electrons (cm2) | 5.0×10^-13^ | 5.0×10^-15^ | 1.0×10^-13^ | 1.0×10^-13^ |
| Capture cross section holes (cm2) | 1.0×10^-15^ | 1.0×10^-13^ | 1.0×10^-15^ | 1.0×10^-15^ |
| Energetic distribution | Single | Single | Single | Single |
| Reference for defect energy level Et | Above EV | Above EV | Above EV | Above EV |
| Energy level with respect to reference (eV) | 1.650 | 1.650 | 1.20 | 0.8 |
| Deep defect density (cm-3) | 1.8×10^16^ | 5.0×10^14^ | 6.0×10^17^ | 4.0×10^14^ |
Supporting Information

## Slide 5
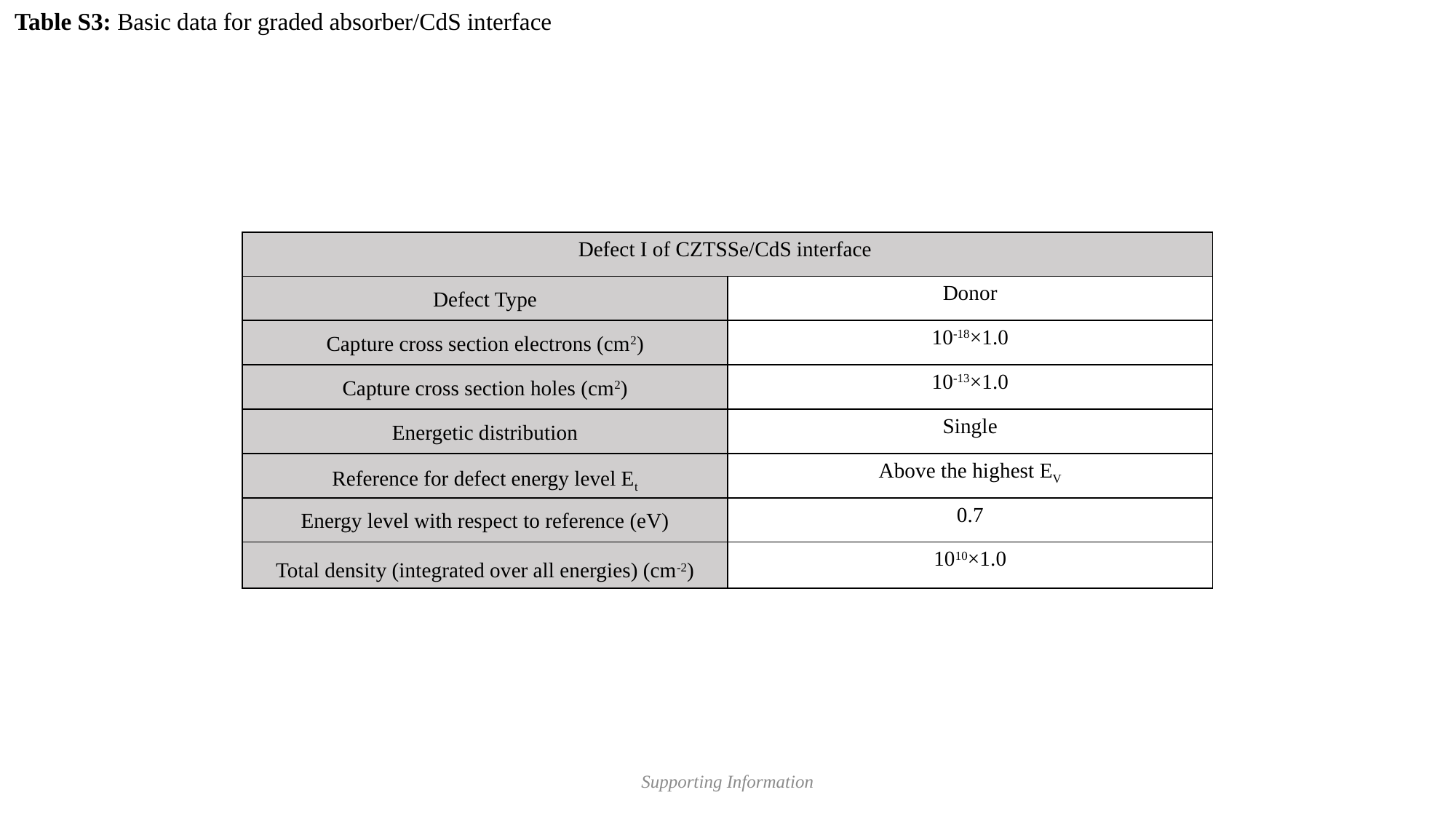

Table S3: Basic data for graded absorber/CdS interface
| Defect I of CZTSSe/CdS interface | |
| --- | --- |
| Defect Type | Donor |
| Capture cross section electrons (cm2) | 1.0×10^-18^ |
| Capture cross section holes (cm2) | 1.0×10^-13^ |
| Energetic distribution | Single |
| Reference for defect energy level Et | Above the highest EV |
| Energy level with respect to reference (eV) | 0.7 |
| Total density (integrated over all energies) (cm-2) | 1.0×10^10^ |
Supporting Information

## Slide 6
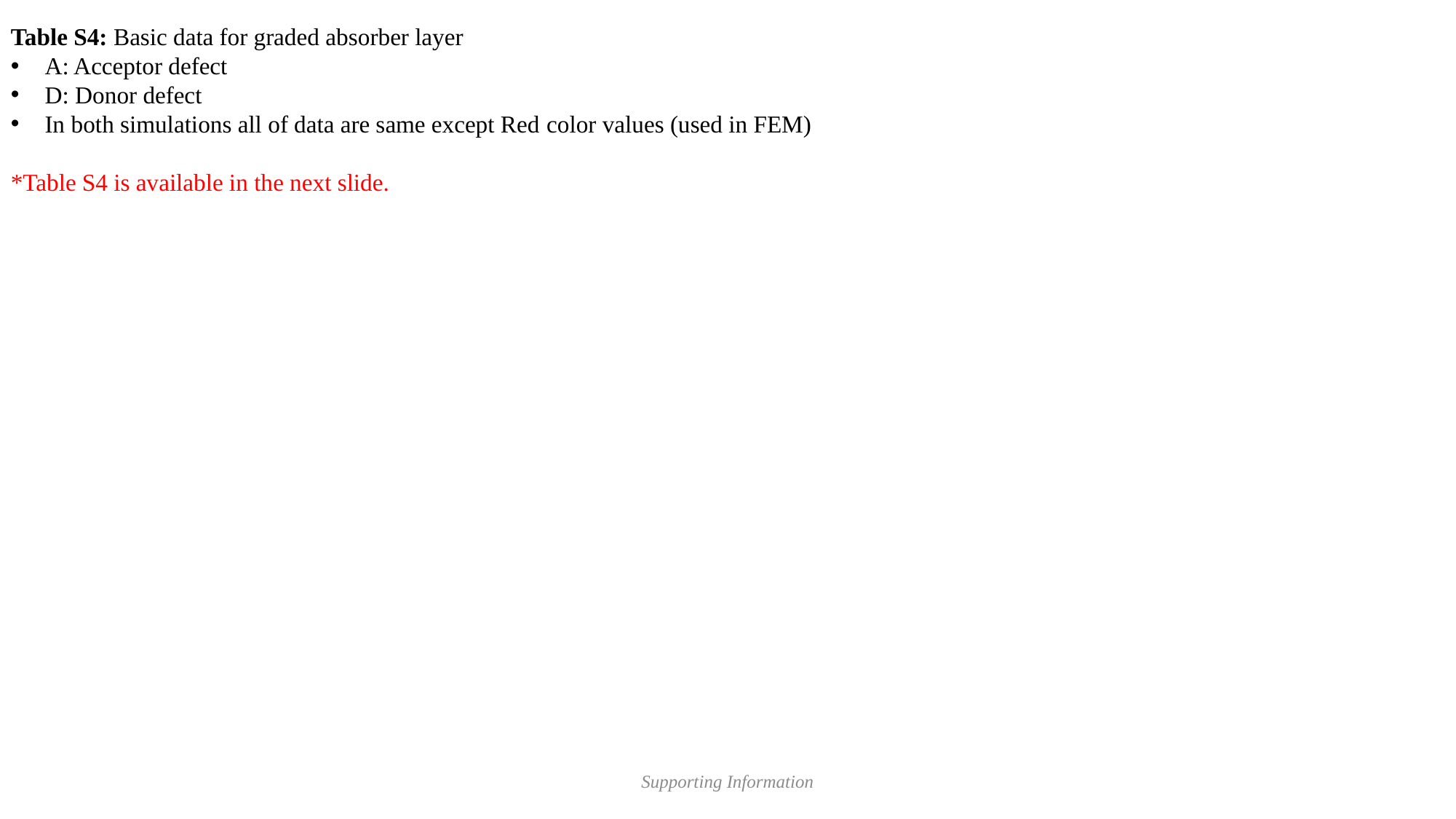

Table S4: Basic data for graded absorber layer
A: Acceptor defect
D: Donor defect
In both simulations all of data are same except Red color values (used in FEM)
*Table S4 is available in the next slide.
Supporting Information

## Slide 7
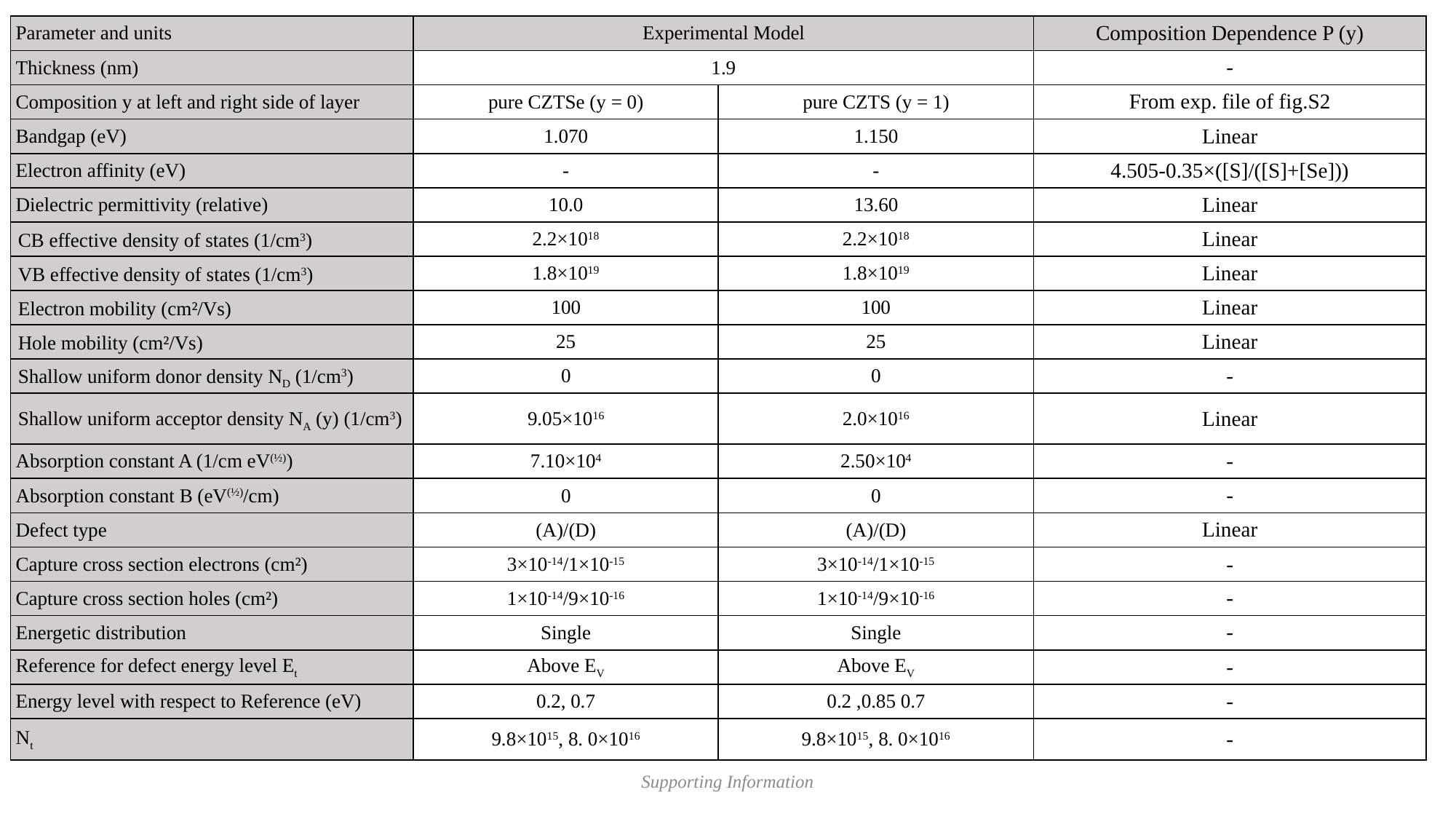

| Parameter and units | Experimental Model | | Composition Dependence P (y) |
| --- | --- | --- | --- |
| Thickness (nm) | 1.9 | | - |
| Composition y at left and right side of layer | pure CZTSe (y = 0) | pure CZTS (y = 1) | From exp. file of fig.S2 |
| Bandgap (eV) | 1.070 | 1.150 | Linear |
| Electron affinity (eV) | - | - | 4.505-0.35×([S]/([S]+[Se])) |
| Dielectric permittivity (relative) | 10.0 | 13.60 | Linear |
| CB effective density of states (1/cm3) | 2.2×10^18^ | 2.2×10^18^ | Linear |
| VB effective density of states (1/cm3) | 1.8×10^19^ | 1.8×10^19^ | Linear |
| Electron mobility (cm²/Vs) | 100 | 100 | Linear |
| Hole mobility (cm²/Vs) | 25 | 25 | Linear |
| Shallow uniform donor density ND (1/cm3) | 0 | 0 | - |
| Shallow uniform acceptor density NA (y) (1/cm3) | 9.05×10^16^ | 2.0×10^16^ | Linear |
| Absorption constant A (1/cm eV(½)) | 7.10×10^4^ | 2.50×10^4^ | - |
| Absorption constant B (eV(½)/cm) | 0 | 0 | - |
| Defect type | (A)/(D) | (A)/(D) | Linear |
| Capture cross section electrons (cm²) | 3×10-14/1×10-15 | 3×10-14/1×10-15 | - |
| Capture cross section holes (cm²) | 1×10-14/9×10-16 | 1×10-14/9×10-16 | - |
| Energetic distribution | Single | Single | - |
| Reference for defect energy level Et | Above EV | Above EV | - |
| Energy level with respect to Reference (eV) | 0.2, 0.7 | 0.2 ,0.85 0.7 | - |
| Nt | 9.8×10^15^, 8. 0×10^16^ | 9.8×10^15^, 8. 0×10^16^ | - |
Supporting Information

## Slide 8
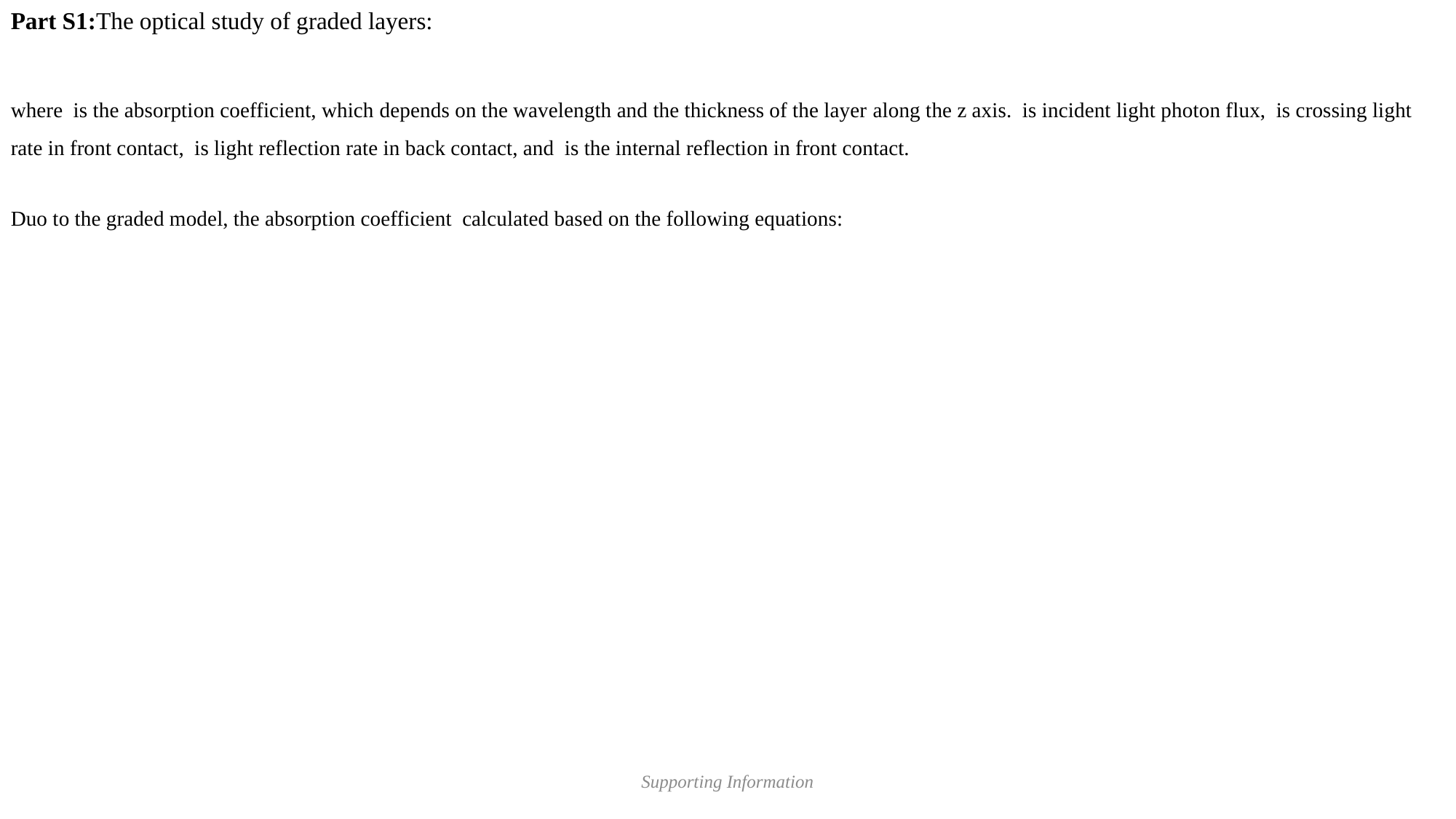

Part S1:The optical study of graded layers:
Supporting Information

## Slide 9
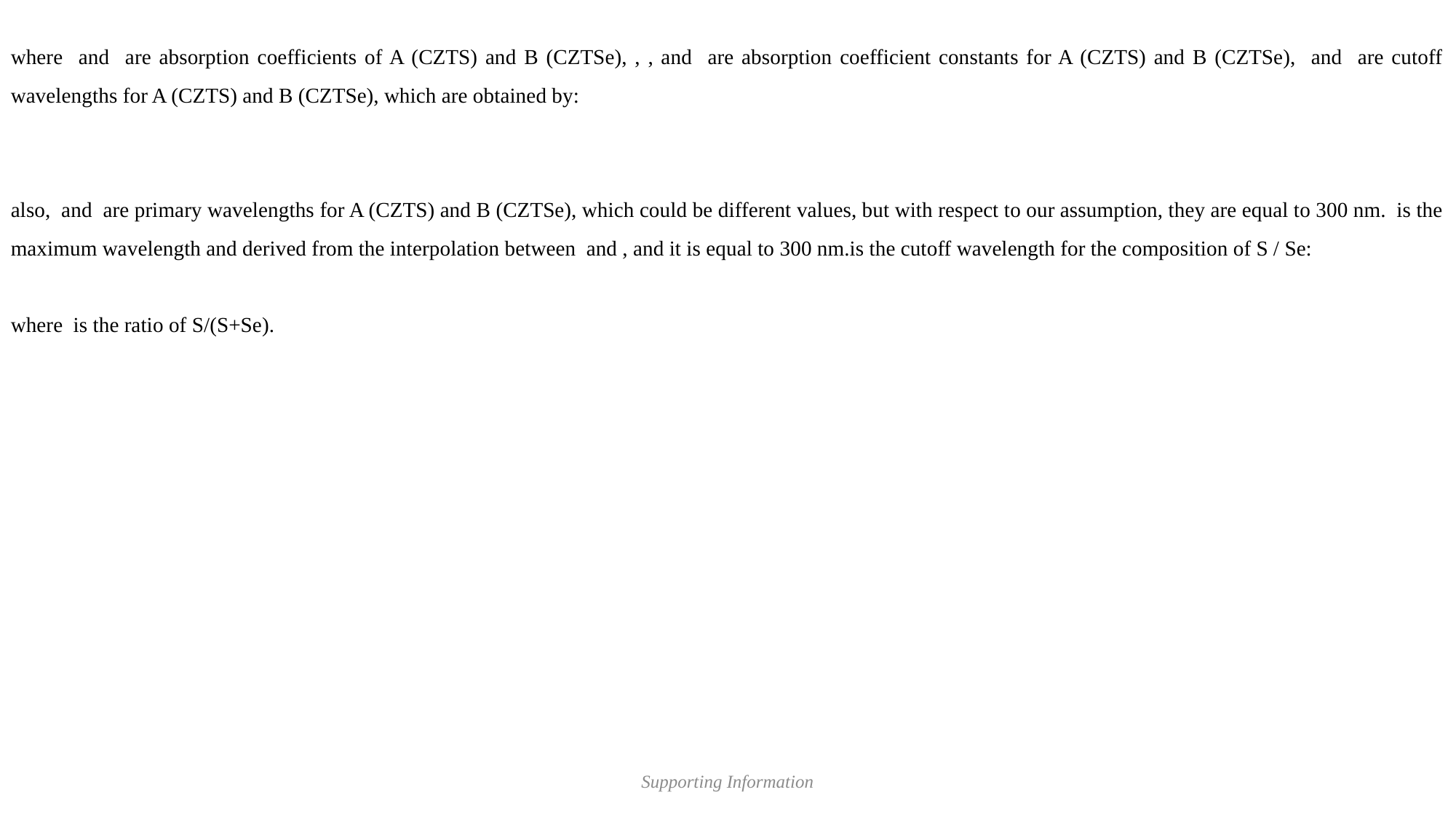

Supporting Information

## Slide 10
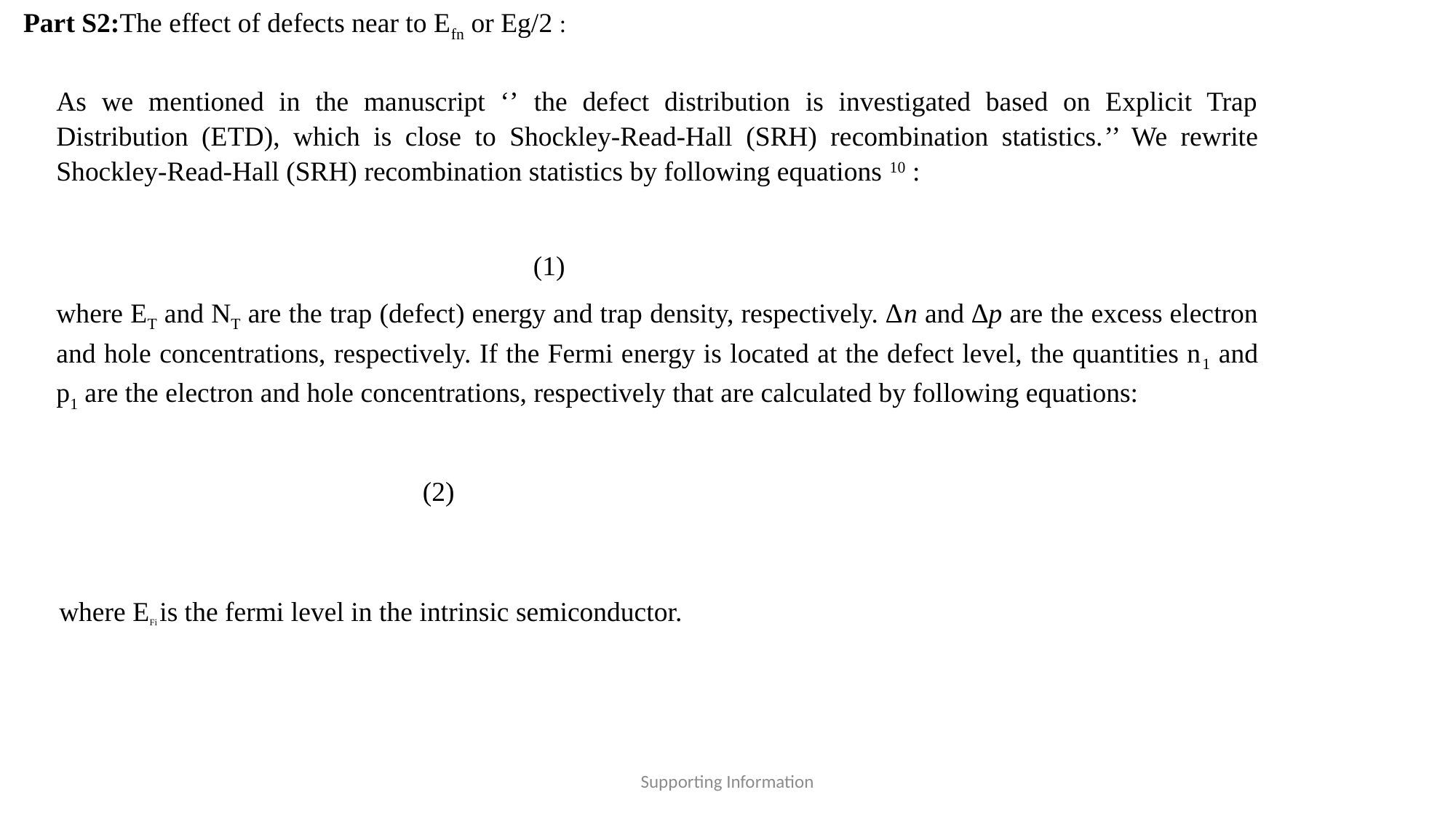

Part S2:The effect of defects near to Efn or Eg/2 :
where EFi is the fermi level in the intrinsic semiconductor.
Supporting Information

## Slide 11
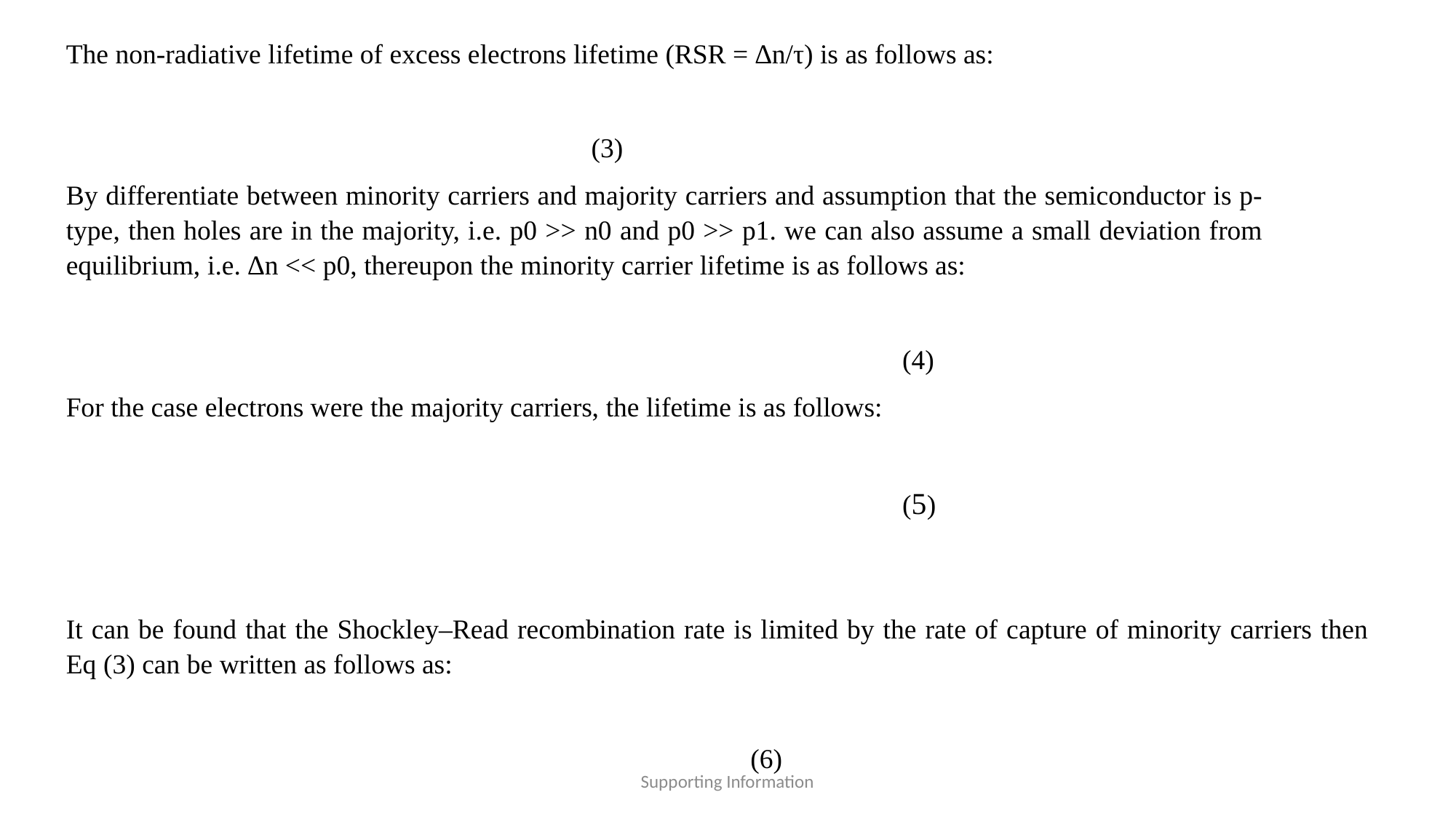

Supporting Information

## Slide 12
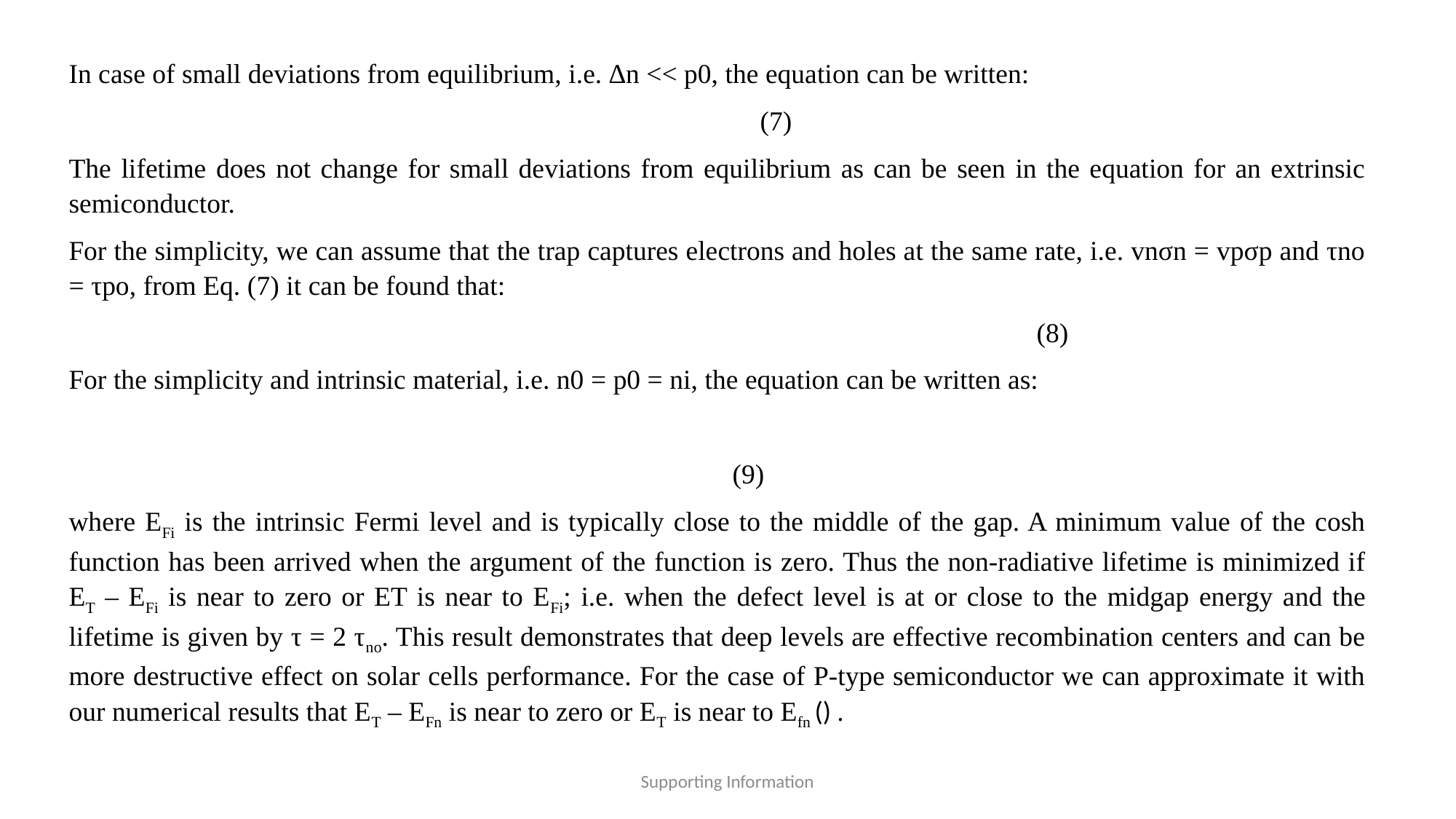

Supporting Information

## Slide 13
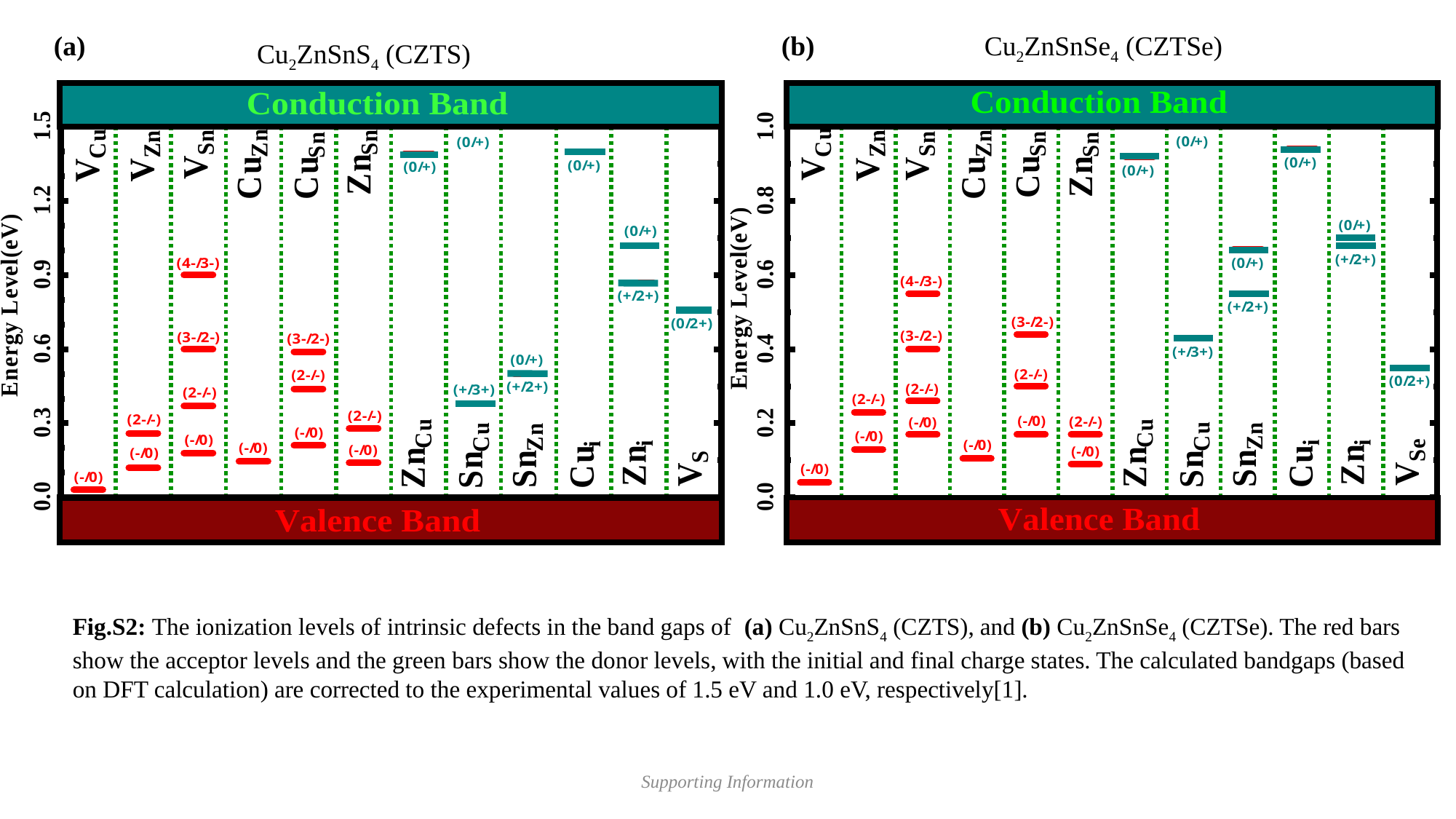

(a)
(b)
Cu2ZnSnSe4 (CZTSe)
Cu2ZnSnS4 (CZTS)
Fig.S2: The ionization levels of intrinsic defects in the band gaps of (a) Cu2ZnSnS4 (CZTS), and (b) Cu2ZnSnSe4 (CZTSe). The red bars show the acceptor levels and the green bars show the donor levels, with the initial and final charge states. The calculated bandgaps (based on DFT calculation) are corrected to the experimental values of 1.5 eV and 1.0 eV, respectively[1].
Supporting Information

## Slide 14
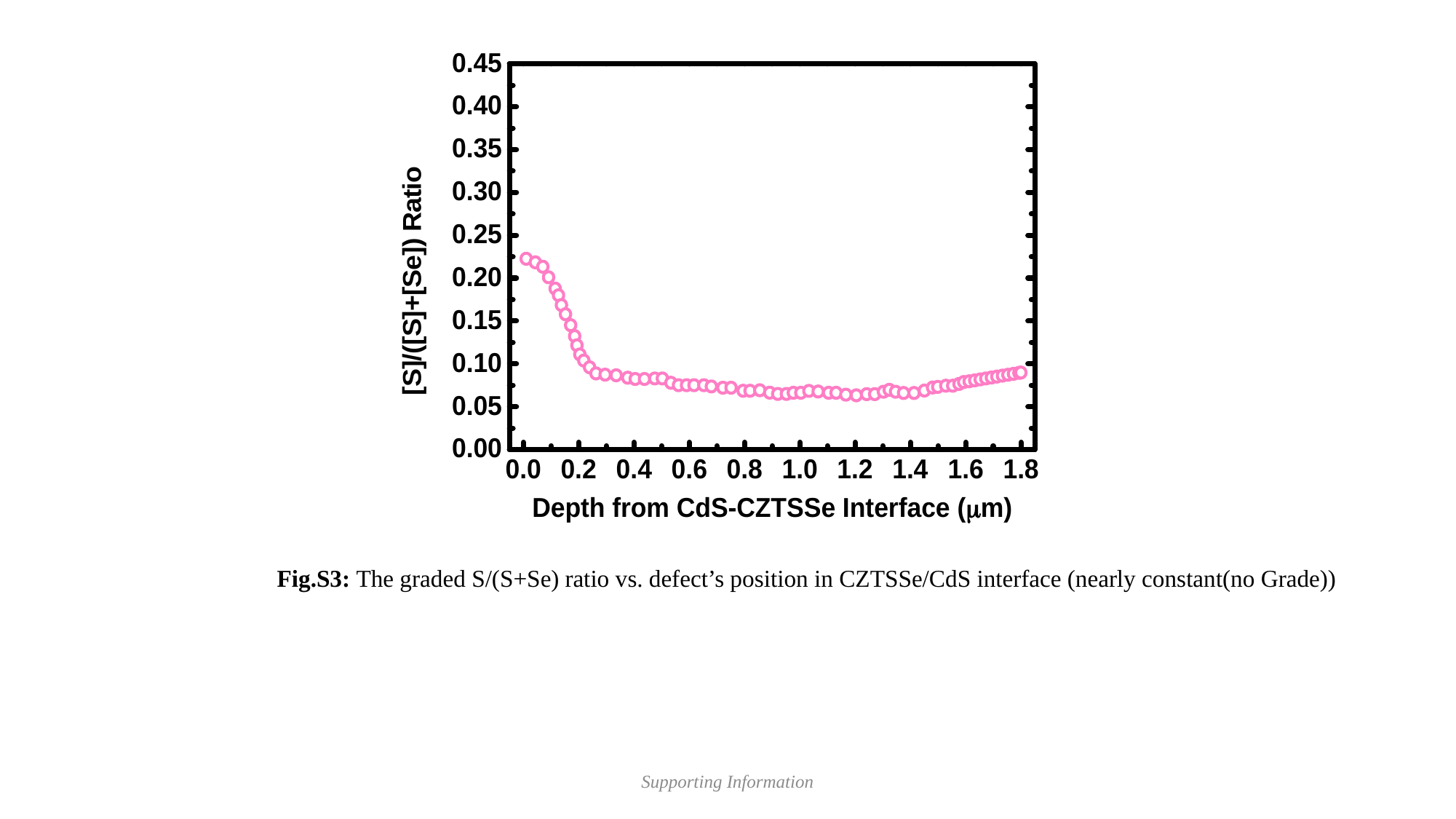

Fig.S3: The graded S/(S+Se) ratio vs. defect’s position in CZTSSe/CdS interface (nearly constant(no Grade))
Supporting Information

## Slide 15
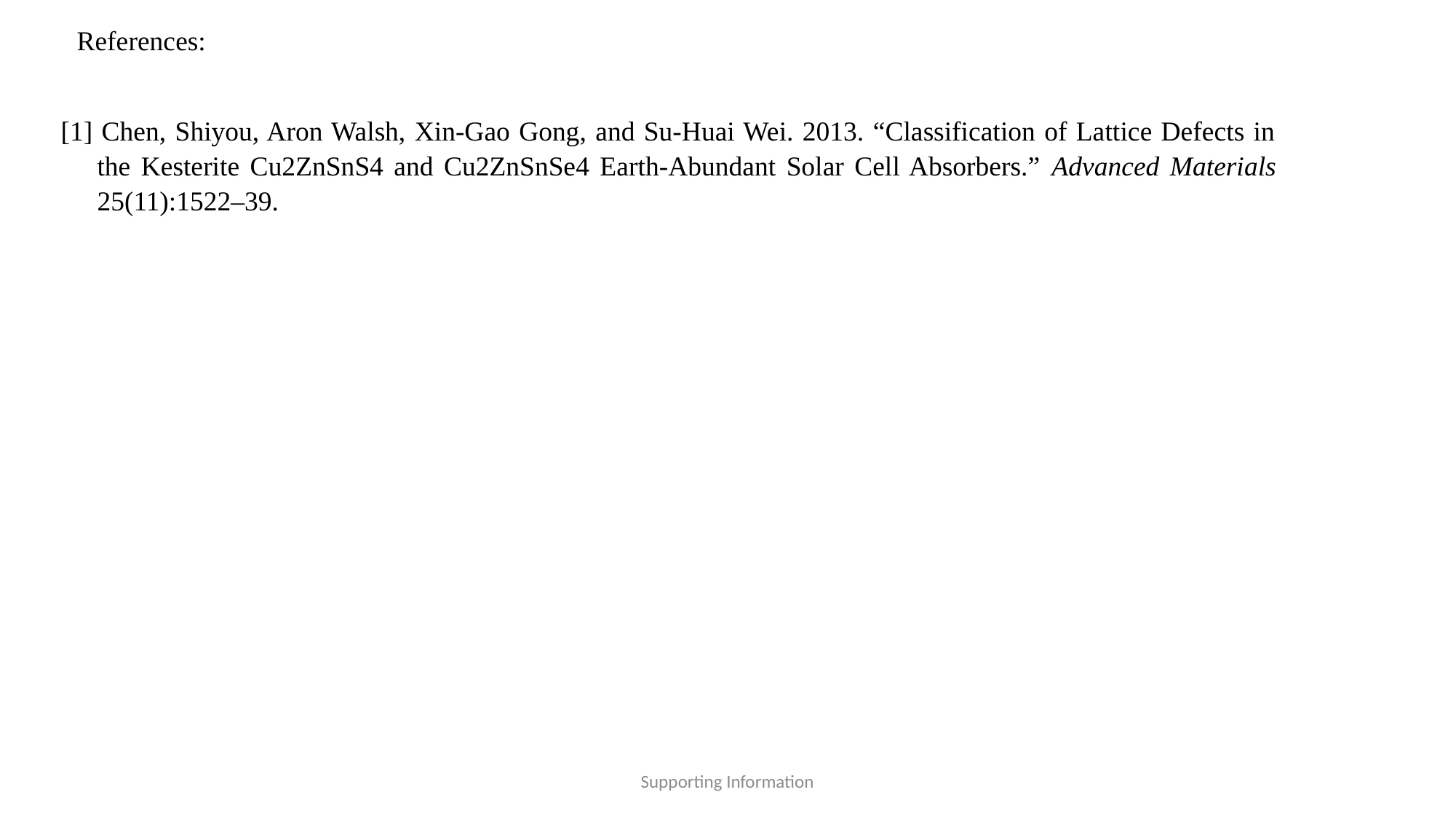

References:
[1] Chen, Shiyou, Aron Walsh, Xin-Gao Gong, and Su-Huai Wei. 2013. “Classification of Lattice Defects in the Kesterite Cu2ZnSnS4 and Cu2ZnSnSe4 Earth-Abundant Solar Cell Absorbers.” Advanced Materials 25(11):1522–39.
Supporting Information
